# NLRP3-mediated pyroptosis in diabetic nephropathy

**DOI:** 10.3389/fphar.2022.998574

**Published:** 2022-10-11

**Authors:** Jiayi Wan, Dongwei Liu, Shaokang Pan, Sijie Zhou, Zhangsuo Liu

**Affiliations:** ^1^ Traditional Chinese Medicine Integrated Department of Nephrology, The First Affiliated Hospital of Zhengzhou University, Zhengzhou, China; ^2^ Research Institute of Nephrology, Zhengzhou University, Zhengzhou, China; ^3^ Henan Province Research Center for Kidney Disease, Zhengzhou, China; ^4^ Key Laboratory of Precision Diagnosis and Treatment for Chronic Kidney Disease in Henan Province, Zhengzhou, China

**Keywords:** diabetic nephropathy, pyroptosis, pathogenesis, signaling pathways, drugs

## Abstract

Diabetic nephropathy (DN) is the main cause of end-stage renal disease (ESRD), which is characterized by a series of abnormal changes such as glomerulosclerosis, podocyte loss, renal tubular atrophy and excessive deposition of extracellular matrix. Simultaneously, the occurrence of inflammatory reaction can promote the aggravation of DN-induced kidney injury. The most important processes in the canonical inflammasome pathway are inflammasome activation and membrane pore formation mediated by gasdermin family. Converging studies shows that pyroptosis can occur in renal intrinsic cells and participate in the development of DN, and its activation mechanism involves a variety of signaling pathways. Meanwhile, the activation of the NOD-like receptor thermal protein domain associated protein 3 (NLRP3) inflammasome can not only lead to the occurrence of inflammatory response, but also induce pyroptosis. In addition, a number of drugs targeting pyroptosis-associated proteins have been shown to have potential for treating DN. Consequently, the pathogenesis of pyroptosis and several possible activation pathways of NLRP3 inflammasome were reviewed, and the potential drugs used to treat pyroptosis in DN were summarized in this review. Although relevant studies are still not thorough and comprehensive, these findings still have certain reference value for the understanding, treatment and prognosis of DN.

## Introduction

Inflammasomes were first discovered in 2002 as multi-protein complexes with the function of inducing inflammation (Martinon et al., 2002). The assembly and activation of inflammasomes can occur in different organelles such as mitochondria, endoplasmic reticulum, and nucleus (Pandey et al., 2021). Clinical diagnosis of certain diseases and monitoring of treatment response can be realized through inflammasome imaging systems (Nandi et al., 2022). NOD-like receptor thermal protein domain associated protein 3 (NLRP3) inflammasome is a well-studied inflammasome, which is mainly composed of NLRP3, apoptosis-associated speck-like protein containing a caspase recruitment domain (ASC) and caspase-1, and assembled after pattern recognition receptors (PRRs) receive danger signals (Broz and Dixit, 2016). The activation of NLRP3 inflammasome not only leads to an inflammatory response, but also induces a type of lytic cell death known as pyroptosis (Huang et al., 2021b).

Over long periods of time, the study of pyroptosis was put on hold and was once defined as “apoptosis” (Zychlinsky et al., 1994; Hersh et al., 1999). It was not until 2015 that Academician Shao Feng and his team reported the process of gasdermin D (GSDMD) being cleaved by caspase family that people began to make new breakthroughs in the study of pyroptosis (Shi et al., 2015). Currently, pyroptosis is defined as a member of programmed cell death (PCD), which has crosstalk between apoptosis and autophagy (Doerflinger et al., 2020; Zhang et al., 2021b). Necroptosis, pyroptosis, and ferroptosis are three widely studied non-apoptotic cell deaths. Molecularly, necroptosis is a form of PCD that depends on the sequential activation of receptor interacting serine/threonine kinase 3 (RIPK3) and mixed lineage kinase domains, which can assemble into oligomeric complexes called necrosomes (Linkermann and Green, 2014). Ferroptosis is characterized by the overwhelming, iron-dependent accumulation of lethal lipid ROS, independent of caspases and necrosomes components (Dixon et al., 2012). Apoptosis involves several different activation mechanisms, including the intrinsic and extrinsic pathways, intrinsic endoplasmic reticulum pathway, and these processes dependent on TNF receptors, caspase-3, and Bcl-2 family (Wong, 2011). Pyroptosis mainly depends on the pore-forming properties of the gasdermin family (Yu et al., 2021b). Morphologically, apoptosis is manifested in the formation of apoptotic bodies, cytoplasmic shrinkage, and chromatin condensation; necroptosis is mainly manifested by cell swelling, and there are no obvious involvement of phagocytes and lysosomes; ferroptosis is mainly manifested by mitochondrial changes, including shrinkage, electron-dense ultrastructure, and reduced/disappeared cristae (Galluzzi et al., 2018). It has been recognized that when pyroptosis occurs, deoxyribonucleic acid (DNA) double-strand breaks, cells swell, pores form in the cell membrane, and cells contents leak out, resulting in the destruction of the balance of sodium and potassium ions inside and outside the cells (Kovacs and Miao, 2017). These different cell death modes perform different functions. Although pyroptosis can recruit immune cells to attack pathogens by releasing inflammatory factors, excessive pyroptosis can damage cell membrane integrity and lead to organ damage (Man et al., 2017). Initially, pyroptosis was thought to occur only in immune cells, but a large number of studies have shown that pyroptosis can also occur in other cell types. For instance, pyroptosis can promote the proliferation, invasion, and metastasis of cancer cells, and it can also induce retinopathy under high glucose (HG) stimulation (Zhou and Fang, 2019; Gan et al., 2020).

Diabetic nephropathy (DN) is not only a chronic disease with a complex pathogenesis, but also one of the main factors leading to end-stage renal disease (ESRD) (Ilyas et al., 2017). Based on current epidemiological data, the number of DN patients is expected to increase further in the coming decades (Saeedi et al., 2019). The occurrence of DN can induce a series of abnormal changes such as glomerular hypertrophy, podocyte loss, and mesangial matrix expansion (Alicic et al., 2017). Progressive DN is not only the result of glucose metabolism disorder and reactive oxygen species (ROS) production, but also the result of chronic low-grade inflammation and fibrosis (Rayego-Mateos et al., 2020; Maiti, 2021). Genomics analysis found significant ferroptosis in the DN group (Wang et al., 2022c). It has also been found that necroptosis shares several upstream signaling pathways with apoptosis, and that necroptosis may have a greater impact on podocyte loss in DN than apoptosis under the regulation of ubiquitin C-terminal hydrolase L1 (UCHL1) (Xu et al., 2019). Furthermore, studies have shown that in the DN mouse model, the redox balance in kidney cells was disrupted and pyroptosis was shown to be triggered, resulting in loss of kidney cells and impaired kidney function (Cuevas and Pelegrín, 2021). The release of a large number of pro-inflammatory factors such as Interleukin-1β (IL-1β) and Interleukin-18 (IL-18) will lead to an increase in renal vascular permeability, and the urinary protein excretion rate will further increase (Yaribeygi et al., 2019).

NLRP3 inflammasome-mediated pyroptosis is one of the main activation mechanisms of pyroptosis and also a key step in the activation of inflammatory responses. When the NLRP3 inflammasome is activated, it converts inactive pro-caspase-1 into active cleaved-caspase-1, which subsequently promotes the production of mature IL-1β and IL-18 and cleaves GSDMD. The N-terminal fragment of GSDMD leads to the formation of membrane pores and induces pyroptosis (Wang and Hauenstein, 2020). Meanwhile, the activation of NLRP3 inflammasome can cause the production of a large number of inflammatory factors, which is the pathogenesis of certain inflammatory diseases including DN (Yang et al., 2021b). Hyperglycemia, hyperlipidemia, and hyperuricemia can all activate the NLRP3 inflammasome, and NLRP3 knockout (KO) can attenuate glomerular hypertrophy, glomerulosclerosis, and mesangial matrix expansion in streptozotocin (STZ)-induced diabetic mice (Qiu and Tang, 2016; Wu et al., 2018). Therefore, targeting NLRP3 and GSDMD to inhibit pyroptosis may serve as a potential therapeutic strategy (Newton et al., 2021). In this review, we first introduced three activation mechanisms of pyroptosis and described the correlation between NLRP3 inflammasome activation and pyroptosis, and then we explored several pathways that may lead to NLRP3 inflammasome activation and its effects in pyroptosis.Finally, some potential therapeutic drugs for pyroptosis in DN were summarized.

## Three molecular mechanisms of pyroptosis

### Canonical inflammasome pathway

Activation of inflammasome and cleavage of gasdermin family are two of the most important processes in the canonical inflammasome pathway. When the recognition of pathogen-associated molecular patterns (PAMPs) and damage-associated molecular patterns (DAMPs) by PRRs was activated by bacteria, viruses and various pathological factors, different inflammasomes were assembled with the participation of adaptor proteins (such as: ASC) and effector proteins (such as: caspase family) (Lin et al., 2020). Subsequently, pro-caspase-1 can be recruited by inflammasomes and activated into cleaved-caspase-1. Cleaved-caspase-1 can activate IL-1β and IL-18. Next, the activated IL-1β and IL-18 can release into the extracellular in a manner independent of the gasdermin family and mediate the inflammatory cascade. However, GSDMD can be cleaved into GSDMD-N-terminal (GSDMD-NT) with pore forming characteristics and GSDMD-C-terminal (GSDMD-CT), which is the key process leading to pyroptosis (Liu et al., 2016b).

The PRR family includes Toll-like receptors (TLRs) and C-type lectin receptors (CLRs), which are mainly located on cell membranes, and NOD-like Receptors (NLRs) and absent in melanoma 2 (AIM2)-like receptors (ALRs), which are mainly located in the cytoplasm (Plato et al., 2015). Different inflammasomes adapt to different activation mechanisms, and their activation signals are diverse, such as ROS generation, endoplasmic reticulum (ER) stress, calcium (Ca^2+^) overload, and nicotinamide adenine dinucleotide phosphate (NADPH) oxidase (NOXs) activation (Xue et al., 2019). In general, the activation of the NLRP3 inflammasome requires the participation of ASC, but for NLR family CARD domain containing 4 (NLRC4) and NLRP1, they can directly interact with caspase-1 independent of ASC (Zhai et al., 2017; Duncan and Canna, 2018). Additionally, recent studies have shown that Cd exposure can activate AIM2 by increasing oxidative stress (Zhou et al., 2022). Cerebral ischemia/reperfusion (I/R) injury can also induce the release of ectopic dsDNA to promote AIM2 inflammasome assembly and pyroptosis (Li et al., 2019). Moreover, AIM2 has also been identified as a direct target of miR-485, which can inhibit inflammatory response under the action of MEG3 (Liang et al., 2020). It is known that AIM2 inflammasome can also be activated in macrophages to induce GSDMD-dependent pyroptosis (Gao et al., 2019). Interestingly, the expression of GSDMD-N was also significantly increased accompanied by the increase of AIM2 in kidney induced by aldosterone, which aggravated the renal fibrosis (Wu et al., 2022b). Downregulation of AIM2 can reduce the expression of caspase-1, IL-1β, and IL-18 in human glomerular mesangial (HGM) cells (Zhen et al., 2014). Although the above experiments can prove that the AIM2 inflammasome is related to pyroptosis, there is still a gap in the research on whether AIM2 can be a new target for inhibiting pyroptosis in DN.

In addition to AIM2 inflammasome, TLR2, and TLR4 have recently been found to further activate NLRP3 inflammasome by activating nuclear-factor κB (NF-κB) signaling pathway to regulate ozonation-induced pyroptosis (Tian et al., 2021). Meanwhile, the regulation of TLR/NF-κB pathway can also improve renal function and promote renal injury repair (Wang et al., 2019a; Wu et al., 2019). Additionally, Mincle is a C-type lectin receptor whose activation has been shown *in vitro* to promote the release of pro-inflammatory cytokines and pyroptosis of macrophages (Gong et al., 2020). Inhibition of the Mincle/Syk/NF-κB signaling pathway can also reduce the expression of ASC and caspase-1 (He et al., 2022). Although there is still a lack of research on the role of CLRs and TLRs in pyroptosis of DN, their effects on pyroptosis in other cells should not be ignored.

Gasdermin family includes GSDMA, GSDMB, GSDMC, GSDMD, DFNA5, and DFNB59, they are widely expressed in different cells and tissues (Kovacs and Miao, 2017). It has been reported that the N-terminal domain of gasdermin family can bind to phosphorylated phosphatidylinositol and may form pores on lipid membranes (Ding and Shao, 2018). Although such pore formation character of gasdermin family is the molecular basis for pyroptosis, GSDMD is the primary molecule with pore-forming properties in the canonical inflammasome pathway (Ding et al., 2016). And so far, there are no known mechanisms, other than cleavage, for regulating GSDMD (Gao et al., 2022a). GSDME can also mediated pyroptosis, but whether other proteins of gasdermin family can regulate pyroptosis in DN still lacks specific research (Rogers et al., 2019; Li et al., 2021g).

### Non-canonical inflammasome pathway

In the non-canonical inflammasome pathway, caspase-4/5/11 can directly respond to the pathogen structural molecules [e.g., lipopolysaccharide (LPS), lipid A] through the caspase recruitment domain (CARD) and lead to the cleavage of GSDMD and the release of IL-1β and IL-18 (Rathinam et al., 2019). The product of this pathway can also induce the activation of caspase-1 and promote IL-1β and IL-18 maturation (Downs et al., 2020). It has been reported that GSDMB does not induce pyroptosis through its N-terminal like other proteins of gasdermin family, but promotes caspase-4 activity by directly binding to the CARD domain of caspase-4 (Chen et al., 2019b). Subsequently, activated caspase-4 can lead to membrane pore formation with the help of GSDMD, followed by potassium influx to activate NLRP3 (Linder and Hornung, 2020). Downregulation of caspase-4 can inhibit the occurrence of TNF-α-induced pyroptosis of human pulmonary artery endothelial cells (HPAEC) and the activation of GSDMD and GSDME (Wu et al., 2022a). Additionally, leishmania lipophosphoglycan (LPG) and C/EBP homologous protein (CHOP) have also been reported to induce the activation of caspase-11, and activated caspase-11 can directly act on pro-caspase-1 and activate it (Yang et al., 2014; de Carvalho et al., 2019). Caspase-11 has a special recognition mechanism for protein substrates, which is mainly mediated by the P1′-P4′ region of its substrate GSDMD, and caspase-4 and caspase-5 are also regulated by the same mechanism (Bibo-Verdugo et al., 2020). Arginine adenosine-5′-diphosphoribosylation (ADP-ribosylation) of caspase-4/11 can block its recognition and cleavage of GSDMD (Li et al., 2021k). Moreover, CHOP silencing significantly reduced the activity of caspase-11 and the cleavage of GSDMD in renal tubular epithelial cells (Yang et al., 2014; Zhang et al., 2018). The above evidence suggests that inhibiting the activation of non-canonical inflammasome pathway may be a therapeutic target for certain diseases, but this is beyond the scope of this paper. Taking together, the activation of pyroptosis mediated by caspase-4/5/11 has a complex mechanism, and its role in DN still needs to be further explored.

### Caspase-3-mediated inflammasome pathway

**FIGURE 1:**
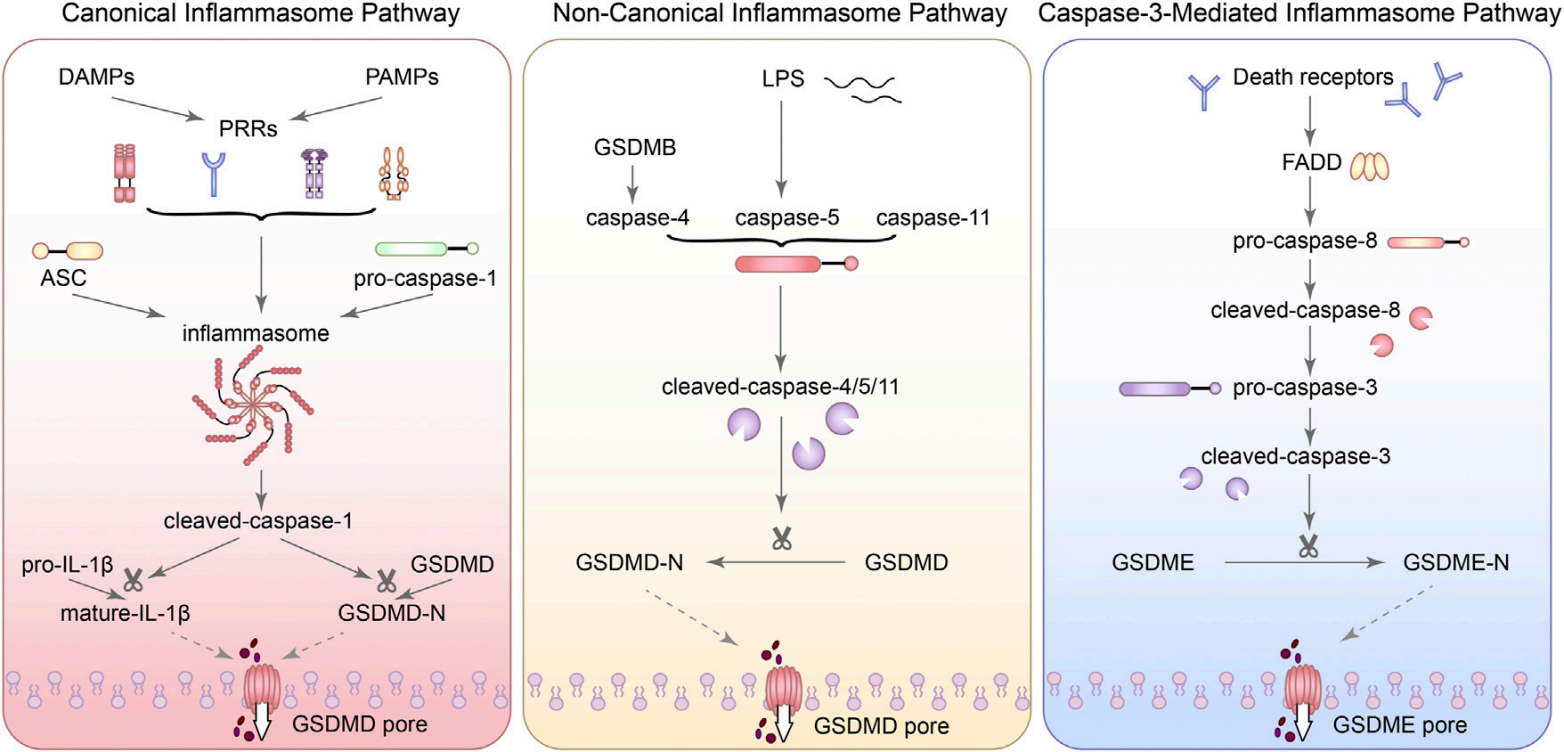
The three molecular mechanisms of pyroptosis. (1) In the canonical inflammasome pathway, when the recognition of PAMPs and DAMPs by PRRs was activated, different inflammasomes were assembled with the participation of adaptor proteins (such as: ASC) and effector proteins (such as: caspase family). Subsequently, pro-caspase-1 can be recruited by inflammasomes and activated into cleaved-caspase-1. Cleaved-caspase-1 can activate IL-1β. Next, the activated IL-1β can release into the extracellular. GSDMD can be cleaved into GSDMD-N-terminal (GSDMD-NT) with pore forming characteristics, which is the key process leading to pyroptosis. (2) In the non-canonical inflammasome pathway, caspase-4/5/11 can directly respond to LPS and lead to the cleavage of GSDMD. GSDMB can promote caspase-4 activity by directly binding to the CARD domain of caspase-4. (3) In the caspase-3-mediated inflammasome pathway, GSDME can also be cleaved by caspase-3 and lead to pyroptosis.

Traditionally, caspase-3 is the core molecular of apoptosis and caspase-8 is also involved in apoptosis. However, recent studies have shown that caspase-3 was activated during LPS-induced pyroptosis (Hu et al., 2021). In some cases, gasdermin E (DFNA5) can also be cleaved by caspase-3 and lead to a transition from apoptosis to pyroptosis (Wang et al., 2017; Zhang et al., 2021h). Although inflammasome is generally considered to play a major role in the canonical inflammasome pathway, AIM2 inflammasome has recently been found to activate caspase-3 and promote DFNA5 expression (Li et al., 2021i). Zeng et al. (2019) also used NLRP3 specific inhibitors to inhibit the activation of the NLRP3-mediated pyroptosis, finding that ATP induced macrophage pyroptosis *via* the caspase-3/GSDME axis. Meanwhile, activated caspase-3 can also inactivate pore-forming domain (PFD) in GSDMD, inhibiting GSDMD-mediated pyroptosis (Taabazuing et al., 2017). It is worth noting that both GSDMD-NT and GSDME-NT can act on mitochondria and make them generate a large amount of ROS, inducing apoptosis and further stimulating the release of inflammatory substances (Rogers et al., 2019). Besides, caspase-1, which mediates the canonical inflammasome pathway, can not only activate caspase-3/7, but also activate Bid in GSDMD deficient cells and lead to apoptosis by inducing the release of cytochrome C in mitochondrial (Tsuchiya et al., 2019). When caspase-11 is overexpressed, caspase-3 can also be activated to promote apoptosis (Miao et al., 2018). Notably, transfection of podocytes with GSDMD siRNA reversed HG-induced inflammation and apoptosis, which may be related to the blocking of JNK signaling pathway (Li et al., 2021a). Although apoptosis-related proteins were also activated in caspase-3-dependent pyroptosis signaling pathway, pyroptosis activation occurred more rapidly (Tsuchiya, 2021). Interestingly, the expression of autophagy-related proteins light chain (LC) 3 I/II and beclin 1 were also reduced with the inhibition of NLRP3 in podocytes of DN (Hou et al., 2020b). Z-DEVD-FMK, an inhibitor of caspase-3, has also been reported to improve proteinuria and tubulointerstitial fibrosis in DN mice and this nephroprotective effect may be related to the inhibition of GSDME (Wen et al., 2020). Moreover, the studies also showed that programmed cell death-ligand 1 (PD-L1) can activate caspase-8 and specifically cleave GSDMC under the action of TNF-α, and then the pores can form in the cell membrane and the apoptosis can transform into pyroptosis (Hou et al., 2020a). Consequently, there is a crosstalk between apoptosis and pyroptosis and even autophagy. Although the mechanism is not completely clear, it provides a new perspective for understanding PCD. Figure 1 summarized the three molecular mechanisms of pyroptosis.

## NLRP3 inflammosome activation and pyroptosis

NLRP3 is a member of the NLRs protein family and also one of the core molecules in the NLRP3 inflammasome. Different domains contained in NLRP3 play different functions. When a leucine-rich repeat (LRR) domain located at the C-terminus is recognized by the ligand, the nucleotide-binding oligomerization domain (NOD) located at the center of the molecule can play an oligomerization role, resulting in conformational rearrangement of NLRP3 and exposing the pyrin domain (PYD) or CARD located at the N-terminus, which subsequently activates the biological effects of the corresponding effector molecules (Inohara et al., 2005). Many studies suggest that inhibiting the expression of NLRP3 has a protective effect on cells (Song et al., 2018a; Wang et al., 2020a). However, it has also been shown that in the unilateral ureter obstruction (UUO) model, Nlrp3−/− increased the damage of renal tubular epithelial cells, and they also suggest that NLRP3 may have an inflammasome-independent role (Pulskens et al., 2014). The reasons for this discrepancy in findings are not fully understood. However, some studies on inflammasome activation did not select cell-specific NLRP3 knockout mice, which may interfere with experimental results due to the inability to distinguish the inflammasomes in the renal parenchyma or phagocytes. It has also been suggested that NLRP3 exerts its function independently of the inflammasome or performs its regulatory role in the form of the NLRP3 inflammasome may be related to different cell types (Komada and Muruve, 2019).

Conventional studies suggest that NLRP3 inflammasome activation is an essential part of the canonical inflammasome pathway. The activation of NLRP3 inflammasome can lead to the cleavage of GSDMD and lytic cell death (Yu et al., 2021b). However, through recent studies in macrophages, Evavold et al. (2018) suggested that GSDMD activation following activation of the NLRP3 inflammasome may determine two distinct cell fates. One induces GSDMD-dependent pyroptosis, while the other induces the indirect release of IL-1 triggered by hyperactivation of cells in a viable state. In other words, even if the NLRP3 inflammasome causes GSDMD to be cleaved by activated caspase-1, pyroptosis does not necessarily occur. Not only that, they also found that the activation of the NLRP3 inflammasome in these two different cell fates is sensitive to different treatments, such as high extracellular potassium concentrations. Meanwhile, several studies in recent years have shown that cytokines can be released from living cells without pyroptosis occurring in the process (Gaidt et al., 2016; Zanoni et al., 2016). Interestingly, GSDMD may also regulate the NLRP3 inflammasome through miR-223 (Kong et al., 2022). Furthermore, in the absence of GSDMD, sustained exposure to LPS can trigger pyroptosis and the release of inflammatory cytokines *via* activation of the caspase-3/GSDME axis. This means that inhibition of GSDMD alone does not completely prevent cytokine secretion and pyroptosis in response to certain inflammatory challenges (Wang et al., 2021a). Consequently, although the advantages and disadvantages of NLRP3 inflammasome blockers, the inhibition of GSDMD-induced pyroptosis and the status of clinical trials have been discussed, the protective effect on cells by inhibiting the activation of the NLRP3 inflammasome is not necessarily related to the inhibition of pyroptosis (Coll et al., 2022). Therefore, when exploring the role of NLRP3 inflammasome inhibitors, it is necessary to verify whether the pyroptosis-associated proteins (such as GSDMD) are regulated accordingly, so as to more accurately understand whether the therapeutic effects of these inhibitors are also related to the regulation of pyroptosis. Despite the many advances made in recent years, the focus on NLRP3 and pyroptosis still leaves gaps in our understanding of the inflammasome. In conclusion, although the activation of the NLRP3 inflammasome does not necessarily lead to pyroptosis, it is still important to review the regulatory pathway of the inflammasome and its potential clinical application as a pre-signal for the activation of the canonical inflammasome pathway of pyroptosis.

## NLRP3 inflammosome-dependent pyroptosis regulatory pathways in diabetic nephropathy

It is well known that chronic sterile inflammation in DN is closely related to renal impairment. The inflammatory cascade induced by NLRP3 inflammasome and IL-1β and IL-18 also has a significant impact on the development of DN. Meanwhile, activation of NLRP3 inflammasome is also the key process to initiate pyroptosis. There are multiple recognized mechanisms or pathways for the activation of NLRP3 inflammasome, such as the massive production of mitochondrial ROS, the reduction of intracellular potassium concentration, and the destabilization of lysosomes (Jo et al., 2016; Paik et al., 2021). However, it still remains to be demonstrated whether the protective effects of these signaling pathways by regulating NLRP3 inflammasome activation are related to pyroptosis. Here we describe several possible signaling pathways, hopefully providing a more complete overview.

### NF-κB/NLRP3 inflammasome signalling pathway

In general, NF-κB binds with inhibitor of NF-κB (IκB) to anchor in cytoplasm in inactive form, dissociates upon stimulation and exposes the active form of P50/P65 heterodimer. It then enters the nucleus and participates in various reactions (Porta et al., 2020). NF-κB can be activated by a variety of factors, and it can regulate inflammatory response, stress response, pyroptosis and apoptosis (Yu et al., 2020; Yu et al., 2021a; Piao et al., 2021). Recently, evidence from clinical and experimental studies has shown that TLRs can induce sterile tubulointerstitial inflammatory responses through the NF-κB signaling pathway. The NLRP3 inflammasome can be activated by NF-κB during this process, linking the perception of metabolic stress in the DN kidney with the activation of a pro-inflammatory cascade (Tang and Yiu, 2020). In addition, it has also been shown that lncRNA-Gm4419 can directly interact with p50, which can interact with the NLRP3 inflammasome and lead to increased expression of pro-inflammatory cytokines in mesangial cells (MCs) under HG stimulation (Yi et al., 2017). These evidences suggest the existence of the NF-κB/NLRP3 axis in the kidney and its regulatory role in the inflammatory response to DN.

In addition to promoting inflammation, the NF-κB/NLRP3 axis also plays a role in regulating pyroptosis. For example, in renal ischemia-reperfusion injury (IRI), Tisp40-dependent phosphorylation promoted the activation of p65 in tubular epithelial cells (TEC) and triggered GSDMD-mediated pyroptosis (Xiao et al., 2020). In DN, this role in regulating pyroptosis has also been confirmed. For instance, the increased activity of the mammalian target of rapamycin (mTOR) can also promote the activation of NF-κB p65 in podocytes and trigger NLRP3-dependent pyroptosis (Wang et al., 2020d). *In vitro* and *in vivo* experiments demonstrated that the activation of the TLR4/NF-κB signaling pathway also promoted GSDMD expression in tubule cells, although they did not examine whether the NLRP3 inflammasome was activated in this experiment (Wang et al., 2019b). However, in another study, knockdown of lncRNA XIST using lentivirus was found to inhibit NLRP3/GSDMD axis-mediated pyroptosis in HK-2 cells through the microRNA-15b-5p (miR-15b-5p)/TLR4 axis (Xu et al., 2022). Furthermore, Xu et al. (2021b) also constructed a DN mouse model and overexpressed the Forkhead box M1 (FOXM1) which can drive renal tubular regeneration in podocytes. They found that can FOXM1 bind to the Sirtuin 4 (SIRT4) promoter and inhibit the phosphorylation of NF-κB, and then the expression of nephrin (a podocyte marker) was increased while the expression of the NLRP3 inflammasome and cleaved-caspase-1 were decreased. Recently, AMPK was used to inhibit the activation of NF-κB *via* silent mating type information regulation-2 homolog-1 (SIRT1), and the expression of NLRP3, caspase-1, IL-1β, and GSDMD-N were observed to decrease in podocytes of DN mice (Li et al., 2020a). The use of catalpol (Cat) can increase the expression of adenosine 5′-monophosphate (AMP)-activated protein kinase (AMPK) and SIRT1, effectively inhibit pyroptosis in podocytes, and improve the abnormal structure and function in kidney of DN (Chen et al., 2020b). These studies further established a relationship between NLRP3 inflammasome activation and pyroptosis in DN, and also provided a solid theoretical basis for how to regulate pyroptosis and play a protective role by inhibiting the NF-κB/NLRP3 axis.

**FIGURE 2:**
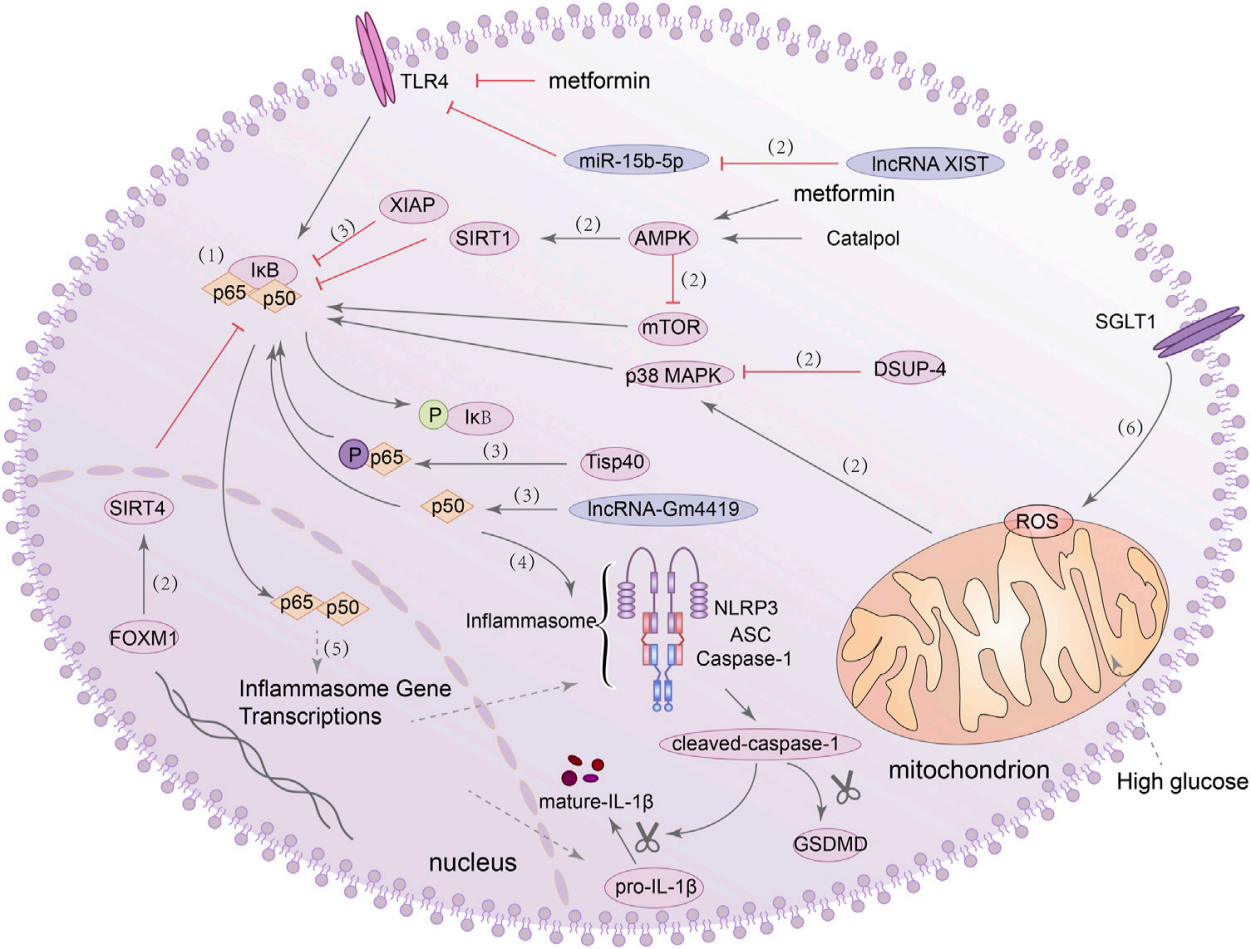
NF-κB/NLRP3 signaling pathway is involved in the regulation of pyroptosis. (1) NF-κB binds with IκB in inactive form, dissociates the active form of P50/P65 heterodimer upon stimulation. (2) lncRNA XIST/miR-15b-5p/TLR4/NF-κB signaling pathway, AMPK/SIRT1/NF-κB signaling pathway, AMPK/mTOR/NF-κB signaling pathway, DSUP-4/MAPK/NF-κB signaling pathway, ROS/MAPK signaling pathway and FOXM1/SIRT4/NF-κB signaling pathway, they all can regulate the activation of NF-κB. (3) Tisp40 and lncRNA-Gm4419 can promote the activation of NF-κB p65/p50, and XIAP can inhibit the activation of NF-κB. (4) P50 can act with NLRP3 inflammasome and lead to increased expression of pro-inflammatory cytokines and GSDMD-N. (5) NF-κB can promote the transcription of inflammasome genes. (6) Knockdown of SGLT1 partially reduced pyroptosis, ROS generation and NF-κB activation.

Recently, Xin et al. (2018) found that X-linked inhibitor of apoptosis protein (XIAP) can also inhibit the activation of NF-κB and alleviate HG-induced podocyte injury and renal fibrosis. Meanwhile, dual specificity phosphatase-4 (DUSP-4) inhibited the sustained activation of p38 and c-Jun N-terminal kinase (JNK) mitogen-activated protein kinase (MAPK) and protected the structure and function of glomeruli and podocytes in DN (Denhez et al., 2019). Inhibition of ROS/MAPK/NF-κB signaling pathway can also inhibit renal dysfunction induced by HG (Chen et al., 2018c). However, inhibition of NF-κB by XIAP or MAPK has not been verified to be related to NLRP3-mediated pyroptosis in the kidney. Interestingly, metformin not only can activate AMPK and inhibit the mTOR pathway to reduce pyroptosis in diabetic cardiomyopathy, but also correct glucose metabolic reprogramming by inhibiting the TLR4/NF-κB signaling pathway and inhibit NLRP3-induced pyroptosis. They also found that downregulation of sodium-glucose cotransporter 1 (SGLT1) also inhibited NF-κB activation and pyroptosis induced by HG (Yang et al., 2019a; Chai et al., 2021; Zhang et al., 2021g). Although it has not been demonstrated that these mechanisms are also present in DN, it is still of interest. In conclusion, these evidences all indicated that the activation of NF-κB/NLRP3 signaling pathway is one of the key factors regulating pyroptosis, and inhibiting the activation of this signaling pathway may inhibit the occurrence of pyroptosis. Figure 2 summarized the functions of different molecules and pathways in NF-κB/NLRP3-mediated pyroptosis.

### TXNIP/NLRP3 inflammasome signalling pathway

Generally speaking, thioredoxin-interacting proteins (TXNIP) can inhibit the antioxidant activity of thioredoxin protein (Trx) when interacting with it (Yoshihara, 2020). In order to prove whether TXNIP has an effect on DN, Qi et al. analyzed the transcription profile of proximal renal tubular epithelial cells under HG condition by using cDNA microarray and found significant changes in TXNIP expression compared with the control group (Qi et al., 2007). Continuous overexpression of TXNIP can lead to the increase of ROS expression and progressive renal interstitial fibrosis (Tan et al., 2015). Knockdown of TXNIP can inhibit phenotypic changes in podocytes and the production of ROS induced by HG by reducing the activity of mTOR signaling pathway (Song et al., 2019). Furthermore, downregulation of TXNIP antagonized EMT induced by HG by inhibiting MAPK activation and transforming growth factor β1 (TGF-β1) expression (Wei et al., 2013). These studies all suggested that TXNIP can be a potential target for the treatment of DN. Considering the phenomenon that has been stated before, that is, increased activity of mTOR signaling pathway can promote the activation of NF-κB and then trigger NLRP3-mediated pyroptosis, and inhibiting the activation of MAPK can also protect renal function by inhibiting the activation of NF-κB, it is not difficult to understand that regulating TXNIP may have a regulatory effect on pyroptosis. Moreover, several studies have shown that TXNIP/NLRP3 axis-dependent pyroptosis can be detected in neuron cells, liver cells, intestinal cells and other cells (Heo et al., 2019; Ding et al., 2020; Jia et al., 2020). It can be seen that TXNIP/NLRP3 signaling pathway also has a regulatory effect on pyroptosis.

**FIGURE 3:**
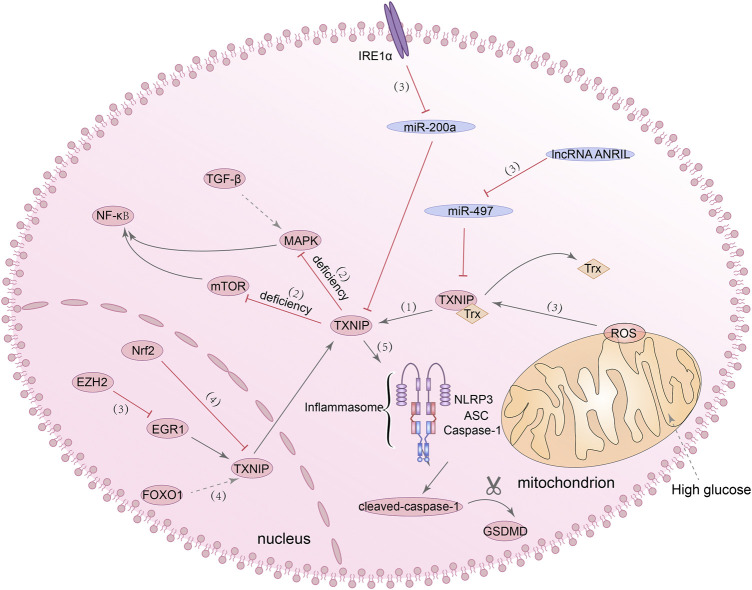
TXNIP/NLRP3 signaling pathway is involved in the regulation of pyroptosis. (1) TXNIP can inhibit the antioxidant activity of Trx. (2) The deficiency of TXNIP can inhibit the MAPK/NF-κB signaling pathway and mTOR/NF-κB signaling pathway. (3) IRE1α/miR-200a/TXNIP signaling pathway, ANRIL/miR-497/TXNIP signaling pathway, mROS/TXNIP signaling pathway and EZH2/EGR1/TXNIP/NLRP3 signaling pathway, they all can regulate the activation of TXNIP. (4) Nrf2 can inhibit the expression of TXNIP, and FOXO1 can promote the expression of TXNIP. (5) The interaction of TXNIP with NLRP3 can promote the activation of NLRP3 inflammasome and lead to increased expression of cleaved-caspase-1 and GSDMD-N.

It has been reported that TXNIP is an NLRP3 binding protein, and its interaction with NLRP3 can promote the activation of NLRP3 inflammasome (Chen et al., 2018b). NLRP3 inflammasome has been found to be activated through mROS/TXNIP/NLRP3 signaling pathway in ischemic acute kidney injury (AKI) (Wen et al., 2018). In the meantime, both *in vitro* and *in vivo* experiments have demonstrated that TXNIP acts as a transcription target of forkhead box O1 (FOXO1). After FOXO1 was knocked out, TXNIP expression and NLRP3 inflammasome activation under HG stimulation were inhibited (Ji et al., 2019; Nyandwi et al., 2020). Besides, inhibition of EZH2/EGR1/TXNIP/NLRP3 signaling pathway and Nrf2/TXNIP/NLRP3 signaling pathway can slow down the progression of DN (Dai et al., 2021; Abd El-Khalik et al., 2022). All of these studies revealed a regulatory role for the TXNIP/NLRP3 axis in DN, but whether NLRP3 inflammasome-dependent pyroptosis is also affected in these processes has not been demonstrated. Of course, in addition to the above-mentioned activation mechanisms of TXNIP/NLRP3 axis, studies have confirmed the other activation mechanisms of TXNIP/NLRP3 axis and their effects on pyroptosis in DN. Wang and Zhao (2021) found that the ANRIL/MIR-497/TXNIP axis was activated in the kidney tissue of diabetic patients, and the high expression of lncRNA-ANRIL can promote the activation of the TXNIP/NLRP3/caspase-1 axis and cause pyroptosis in human kidney-2 (HK-2) cells. ER stress, previously considered as one of the primary culprits leading to the development of DN, has recently been found that one of its pathogenic mechanisms seems to be related to pyroptosis. From *in vitro* experiments to *in vivo*, it has demonstrated that the excessive activation of ER stress sensor inositol-requiring enzyme 1 alpha (IRE1α) can promote the NLRP3-mediated pyroptosis and the expression of TXNIP in renal tubular cells (Ke et al., 2020). Taken together, these evidences suggested that the TXNIP/NLRP3 axis has a role in regulating pyroptosis and is a promising target for the treatment of DN. Figure 3 summarized present studies exploring molecular mechanisms in TXNIP/NLRP3-mediated pyroptosis.

### Nrf2/HO-1/NLRP3 inflammasome signalling pathway

Nuclear factor erythroid 2-related factor 2 (Nrf2) can regulate the intracellular redox balance and has anti-inflammatory effects. Under certain stimulation, Nrf2 will be separated from kelch-like ECH-associated protein 1 (Keap1) and translocated to the nucleus to activate its downstream target genes such as heme oxygenase-1 (HO-1) and superoxide dismutase (SOD) (Bellezza et al., 2018). Activation of Nrf2/HO-1 signaling pathway has been reported to inhibit iron death and apoptosis under HG stimulation (Ma et al., 2020; Antar et al., 2022). Meanwhile, a number of studies in DN mouse model have shown that oxidative stress and inflammatory response in kidney can be improved by activating Nrf2/HO-1 signaling pathway (Alaofi, 2020; Lu et al., 2020). These results suggested that the Nrf2/HO-1 signaling pathway may be a potential target for the treatment of DN.

Recent studies have shown that the Nrf2/HO-1 signaling pathway can also regulate the expression of NLRP3. Chen et al. (2019c) and Huang et al. (2020a) demonstrated the inhibitory effect of activation of Nrf2/HO-1 signaling pathway on NLRP3 inflammatory activation in different cells. Additionally, the increased expression of Nrf2 and HO-1 can also reduce the expression of ROS, caspase-1, and IL-1β (Bian et al., 2020a). Subsequently, one study in alveolar macrophage showed that Nrf2/HO-1 signaling also appears to be involved in NLRP3-mediated pyroptosis (Fei et al., 2020). It has been found that in AKI, inhibition of protein arginine methylation transferase 5 (PRMT5) can significantly reduce the expression of ROS, NLRP3 and GSDMD-N of renal tubular cells by activating the Nrf2/HO-1 signaling pathway (Diao et al., 2019). Besides, inhibition of miR-92a-3p can also alleviate NLRP3-mediated pyroptosis in renal ischemia-reperfusion injury (IRI) *via* its potential target Nrf1 (Wang et al., 2020b). The above studies all indicated that the Nrf2/HO-1 axis was closely related to the production of ROS, and the production of mitochondrial ROS was considered to be a regulator of the NLRP3 inflammasome. However, it has been reported that mitochondrial electron transport chain (ETC) maintains the activation of NLRP3 inflammasome by relying on polymerase chain reaction to generate ATP, but no evidence that mitochondrial ROS is essential for this process has been found (Billingham et al., 2022). Generally speaking, pyroptosis mediated by Nrf2/HO-1/NLRP3 pathway can occur in a variety of cells, which can be considered as a potential therapeutic target.

**FIGURE 4:**
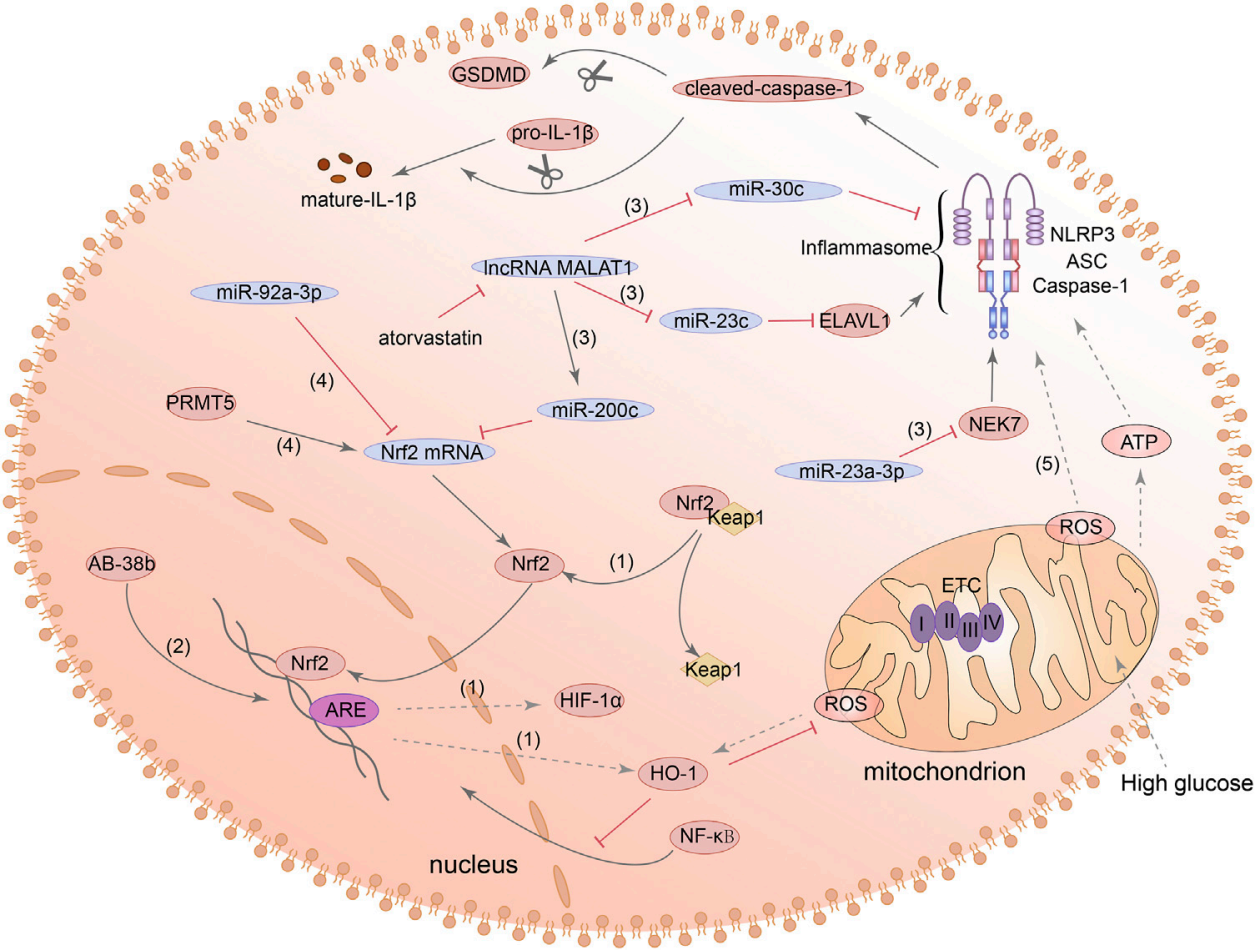
Nrf2/HO-1/NLRP3 signaling pathway is involved in the regulation of pyroptosis. (1) Nrf2 can be separated from Keap1 and translocated to the nucleus to activate its downstream target genes such as HO-1 and HIF-1α. (2) Ab-38b can activate Nrf2 and inhibit NLRP3 expression. (3) lncRNA-MALAT1/miR-200c/Nrf2 signaling pathway, MALAT1/miR-23c/ELAVL1/NLRP3 signaling pathway, MALAT1/miR-30c/NLRP3 signaling pathway and miR-23a-3p/NEK7/NLRP3 signaling pathway, they all can regulate the activation of NLRP3. (4) MiR-92a-3p can inhibit the expression of Nrf2, and PRMT5 can promote the expression of Nrf2. (5) Nrf2 can promote the expression of ROS and ROS can lead to the activation of NLRP3 inflammasome and increase the expression of cleaved-caspase-1 and GSDMD-N.

Recently, the studies on Nrf2/HO-1/NLRP3 signaling pathway and pyroptosis in DN have also made many advances. Ab-38b, which has the property of activating Nrf2, inhibited the expression of NLRP3 and IL-1β by HO-1 in mesangial cells of diabetic mouse. However, this study did not examine whether GSDMD expression was also affected (Du et al., 2020). Other evidence suggested that lncRNA-MALAT1 can act as a molecular sponge, leading to downregulation of miR-30c and promoting the NLRP3-dependent pyroptosis in HK2 cells induced by HG (Liu et al., 2020). Furthermore, MALAT1 can increase the expression of NLRP3 in renal tubular epithelial cells and induce pyroptosis by regulating miR-23c and ELAV like RNA binding protein 1 (ELAVL1) (Li et al., 2017). Other studies in diabetes models found that, miR-23a-3p is also a member of the miR-23 family and can negatively regulate its downstream target gene NEK7 and inhibit NEK7-dependent NLRP3 activation (Zhou et al., 2020; Chang et al., 2021). However, these mechanisms have not been tested in kidney cells. Additionally, miR-200c also has the ability to bind MALAT1 and can affect the expression of Nrf2 and HO-1. Atorvastatin can reduce the overexpression of mir-200c induced by HG in podocytes and inhibit oxidative stress and the expression of NLRP3 and GSDMD-N (Zuo et al., 2021). The above evidences all indicated that the Nrf2/HO-1/NLRP3 signaling pathway has the effect of regulating pyroptosis, but more research is needed to explore the possibility of its use as a therapeutic target in DN. Figure 4 summarized the functions of different molecules and pathways in pyroptosis mediated by Nrf2/HO-1/NLRP3 signaling pathway.

### HIF-1α/NLRP3 inflammasome signalling pathway

Hypoxia inducible factor 1-alpha (HIF-1α) is an active component of HIF-1, which is regulated by hypoxia and regulates the activity of HIF-1 (Yang et al., 2021a). HIF-1α regulates numerous downstream target genes such as vascular endothelial growth factor (VEGF) and glucose transporter-1 (GLUT-1) (Fan et al., 2019; Hepp et al., 2021). Recently, it was found that VEGF, as one of the downstream target genes of HIF-1α, has the effect of inhibiting pyroptosis in hepatocytes (Zhao et al., 2018a). Meanwhile, overexpression of HIF-2α in macrophages improves insulin resistance and reduces NLRP3 inflammasome activation (Li et al., 2021h). Furthermore, various evidences have shown that accumulation of HIF-1α can significantly increase the expression of NLRP3, GSDMD-N, and cleaved caspase-1 in microglia and cardiomyocytes (Jiang et al., 2020; Yuan et al., 2021). Besides, the inhibition of NF-κB/HIF-1α signaling pathway can alleviate hypoxia-induced pyroptosis in C2C12 myoblasts (Yu et al., 2019). These evidences suggested that HIF-1α has a regulatory effect on NLRP3 inflammasome-mediated pyroptosis.

**FIGURE 5:**
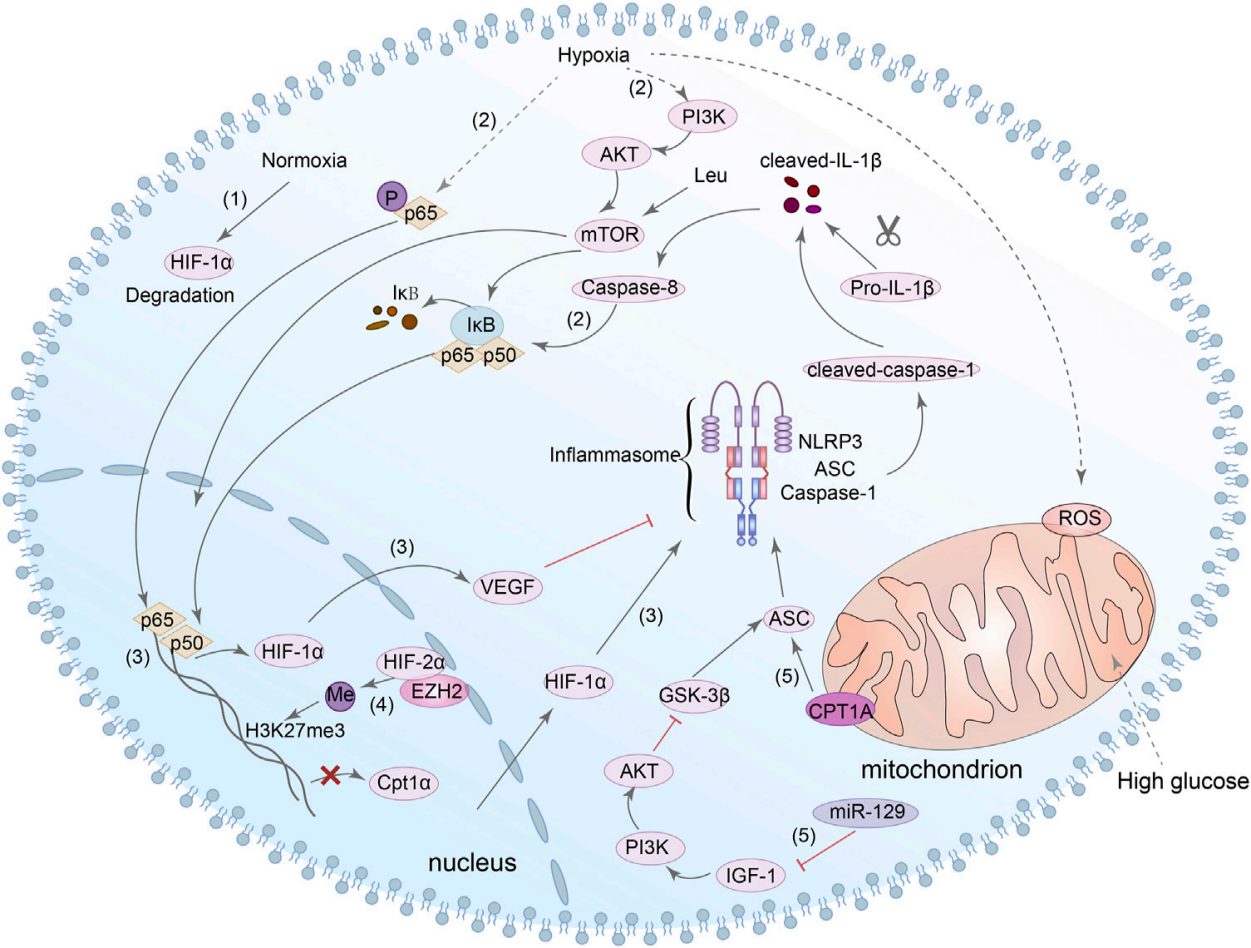
HIF-1α/NLRP3 signaling pathway is involved in the regulation of pyroptosis. (1) HIF-1α can be degraded in normoxia. (2) In hypoxia, NF-κB and PI3K/Akt/mTOR/NF-κB signaling pathway can be activated, and caspase-8 can also promote the nuclear translocation of NF-κB. (3) NF-κB directly bound to the HIF-1α promoter and enhanced its transcription. HIF-1α could modulate the activation of the NLRP3 inflammasome, and the increase in VEGF-A can also result in NLRP3 inflammasome activation. (4) HIF-2α binds directly to the Cpt1a promoter and inhibits the expression of it. HIF-2α can also regulate the H3K27me3 methylation during NLRP3 inflammasome activation. (5) Both Cpt1a and miR-129/IGF-1/PI3K/Akt/GSK3β signaling pathway can regulate the expression of ASC which is involved in the activation of NLRP3 inflammasome.

It is known that HIF-1α is one of the downstream transcription factors of mTOR, and mTOR is one of the downstream targets of PI3K/Akt axis (Appelberg et al., 2020). In the DN mouse model, Leu is an activator of mTOR that can lead to an increase of NLRP3 inflammasome and induce podocytes pyroptosis (Wang et al., 2020d). In addition, caspase-8 can promote the activation of NLRP3 inflammasome by regulating HIF-1α through NF-κB nuclear translocation. Next, mature IL-1β in turn can promote the activation of caspase-8/HIF-1α/NLRP3/NLRP12/NLRC4, resulting in pyroptosis (Huang et al., 2019; Chen et al., 2020a). In conclusion, although it has not been directly demonstrated whether HIF-1α/NLRP3 is related to pyroptosis, we can still speculate that HIF-1α may be a potential target for NLRP3-mediated pyroptosis in DN. Notably, ASC is one of the key components of the activation of NLRP3 inflammasome, and glycogen synthase kinase-3beta (GSK-3β) can interact with ASC and induce the activation of NLRP3 inflammasome in cardiac fibroblast (Wang et al., 2020c). Simultaneously, inhibition of miR-129 can alter the pyroptosis rate of neuronal cells through IGF-1/GSK3β signaling pathway (Wang et al., 2021b). Meanwhile, Wang et al. also found that inhibition of GSK-3β reduced NLRP3 expression and inhibited pyroptosis in cardiomyocytes (Wang et al., 2022b). Interestingly, several studies have shown that GSK-3β can regulate the expression of HIF-1α (Flügel et al., 2007; Mennerich et al., 2014). However, there is no direct evidence of the relationship between HIF-1α, GSK-3β and pyroptosis of renal cells in DN. Consequently, the mechanism between HIF-1α, GSK-3β and NLRP3-dependent pyroptosis in DN needs to be further explored. Figure 5 summarized present studies exploring molecular mechanisms in HIF-1α/NLRP3-mediated pyroptosis.

### PTEN/PI3K/Akt inflammasome signalling pathway

Phosphatase and tensin homologue (PTEN) is a newly discovered tumor suppressor gene, whose protein products have the functions of dephosphorylating and can regulate apoptosis, cell metastasis and cell growth (Khokhar et al., 2020). A recent study found that podocyte dysfunction and proteinuria seem to be related to the reduced expression of PTEN in mouse models of DN (Lin et al., 2015). Another study showed that epithelial-mesenchymal transformation (EMT) of renal tubular epithelial cells induced by HG was also aggravated by the decreased expression of PTEN, and the mechanism may be related to peroxisome proliferator-activated receptor gamma (PPARγ) (Yan et al., 2019). Meanwhile, in a streptozotocin (STZ)-induced diabetic mouse model, upregulation of PTEN can reduce the phosphorylation levels of phosphoinositide 3-kinases (PI3K) and protein kinase B (Akt/PKB), thereby alleviating inflammation and renal interstitial fibrosis in DN (Song et al., 2020). These studies demonstrated the protective effect of PTEN on DN kidney cells. Interestingly, methyltransferase-like protein 3 (METTL3) can modify PTEN, and the increased expression of METTL3 has a specific inhibitory effect on pyroptosis of podocytes under HG condition (Liu et al., 2021a). Meanwhile, experiments in the human retinal pigment epithelium (RPE) cell line and cardiomyocyte also demonstrated that METTL3 overexpression can inhibit PTEN and increase the phosphorylation level of Akt (Zha et al., 2020). Next, activation of SIRT1 can also inhibit ROS generation and NLRP3 inflammasome activation through the activation of Akt signaling pathway (Han et al., 2020). Besides, other studies have also shown that NLRP3-mediated pyroptosis can also be affected by PI3K/Akt pathway in liver cells (Li et al., 2018b). The PTEN/PI3K/Akt signaling pathway will be affected when the level of ROS *in vivo* incresed, resulting in increased expression of NLRP3, caspase-1 and GSDMD-N (Zhang et al., 2021c). It can be seen that the PTEN/PI3K/Akt signaling pathway also has a role in regulating NLRP3-mediated pyroptosis.

**FIGURE 6:**
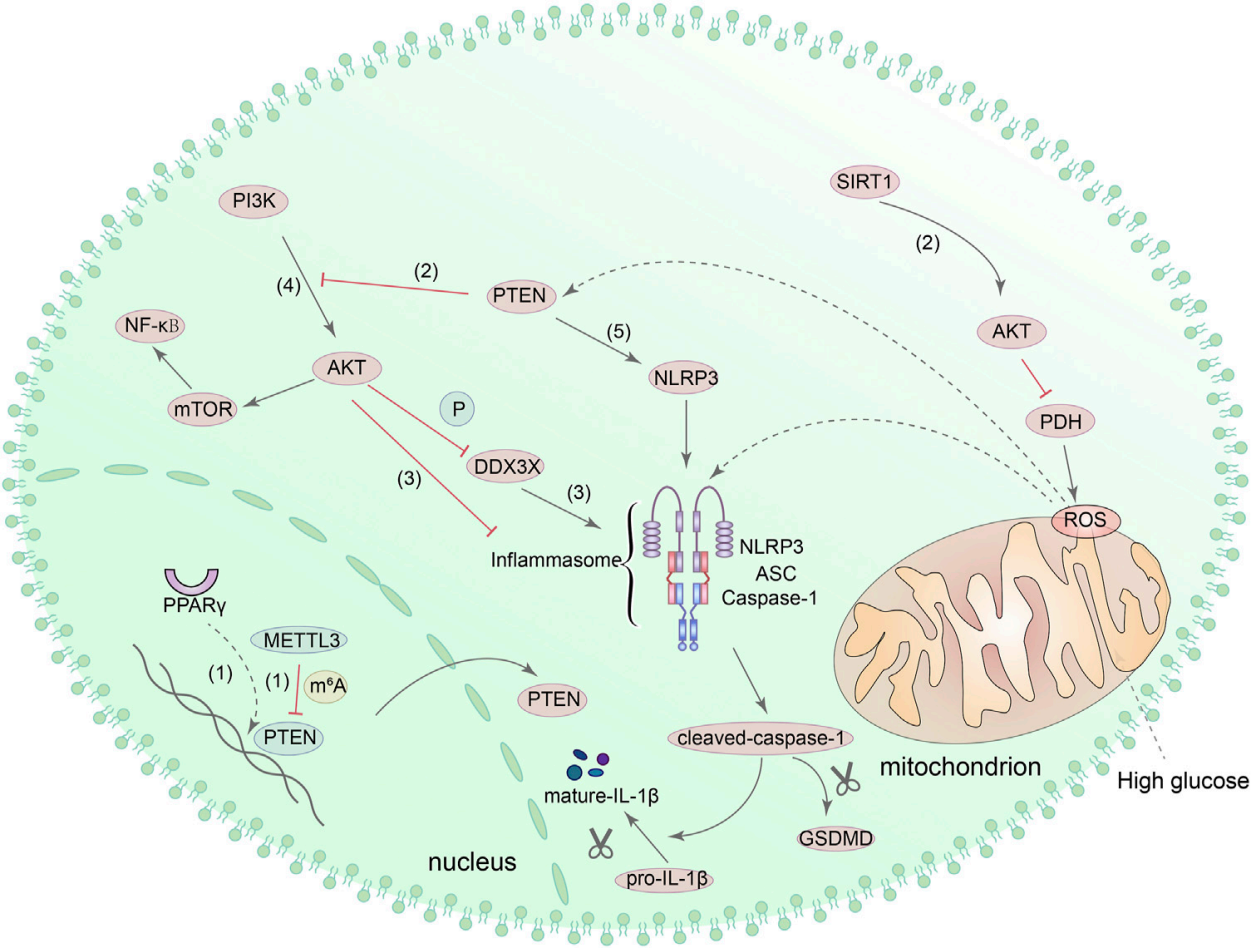
PTEN/PI3K/Akt signaling pathway is involved in the regulation of pyroptosis. (1) PPAR can regulate the transcription of PTEN, and METTL3 can also modify PTEN. (2) Upregulation of PTEN can reduce the phosphorylation of PI3K and Akt. SIRT1 can interact with Akt directly, consequently promoting the activity of Akt and inhibiting the production of ROS *via* Akt/PDH axis. (3) DDX3X can be phosphorylated by Akt, and Akt can increase the phosphorylation of NLRP3. (4) PI3K/Akt/mTOR/NF-κB signaling pathway can also regulate pyroptosis mediated by NLRP3 inflammasome. (5) PTEN directly interacts with NLRP3 and enables NLRP3 inflammasome assembly and activation.

To further investigate the relationship between PTEN and pyroptosis, Zhao et al. (2020) induced macrophages with LPS and found that the activation of NLRP3 was inhibited with Akt by increasing the phosphorylation of NLRP3 at S5 and decreasing the ubiquitination at lysine 496. Furthermore, as a component of NLRP3 inflammasome, DEAD-box helicase 3 X-linked (DDX3X) can be phosphorylated by Akt and affect the function of NLRP3 inflammasome (Guo et al., 2021). These evidences suggested that Akt has a direct role in regulating NLRP3. Moreover, other studies suggested that the activation of mTOR, which is the downstream target of PI3K/Akt, can also promote NLRP3-mediated pyroptosis, and PTEN can directly interact with NLRP3 to activate NLRP3 inflammasome (Huang et al., 2020c; Wang et al., 2020d). However, none of the above-mentioned mechanisms have been confirmed in the DN kidney model, and further verification is required. Figure 6 shows the potential mechanism of pyroptosis mediated by PTEN/PI3K/Akt signaling pathway.

## Pyroptosis and renal cells in diabetic nephropathy

The intrinsic cells of kidney include mesangial cells, capillary endothelial cells, podocytes, renal tubular epithelial cells and renal interstitial fibroblasts. Among them, the glomerular capillary endothelial cells, basement membrane and podocytes constitute the glomerular filtration barrier. The progression of DN is associated with glomerular basement membrane (GBM) thickening, podocytes injury and interstitial fibrosis caused by various factors. In recent years, it has been found that GSDMD and GSDME related to pyroptosis are widely distributed in tissues such as placenta, heart, brain, intestine, and kidney (Ruan et al., 2020). Inhibition of pyroptosis seems to be a new way to delay the development of DN. Here, we reviewed the situation of pyroptosis in podocytes, renal tubular epithelial cells, and kidney endothelial cells.

### Pyroptosis of podocytes in diabetic nephropathy

Because of the notoriously difficult to regenerate, damage to podocytes will lead to damage to the glomerular filtration barrier and the occurrence of proteinuria. Recent studies have shown that the inhibition of pyroptosis as well as the inhibition of apoptosis or autophagy can protect the podocytes structure and function from damage. For instance, combined treatment with mycophenolate mofetil, tacrolimus, and steroids significantly inhibited the activation of NLRP3 and caspase-1 and reduced GSDMD-N levels in lupus nephritis models (Cao et al., 2021). The use of superoxide dismutase-mimic agent and glyburide can also inhibit pyroptosis and NLRP3 inflammatory activation in HIV by inhibiting production of ROS (Haque et al., 2016). Zhang et al. (2021a) also stimulated mouse podocytes with the soluble complement complex C5b-9 and found that a long-chain noncoding RNA (lncRNA) KCNQ1OT1 can interact with miR-486a-3p and induce pyroptosis in podocytes by affecting the transcriptional activity of NLRP3. These results suggested that the inhibition of NLRP3 inflammasome may have a regulatory effect on pyroptosis in podocytes.

In addition to hyperglycemia and other factors involved in the progression of DN, overactivation of pyroptosis in podocytes is also one of the important factors leading to cell loss and dysfunction. Qian et al. demonstrated that excessive activation of pyroptosis mediated by caspase-11/4 and GSDMD in HFD/STZ-induced DN mice can lead to podocyte injury (Cheng et al., 2021). Furthermore, the level of miR-21-5p in exosomes derived from macrophages was increased in response to HG stimulation, and inhibition of miR-21-5p can reduce the production of ROS and the expression of NLRP3, caspase-1 and IL-1β in podocytes (Ding et al., 2021b). In addition, the purinergic P2X7 receptor (P2X7R) expressed in podocytes can lead to the opening of ligand-gated ion channels and potassium outflow in response to ATP stimulation. Next, assembly and activation of NLRP3 inflammasome can be initiated due to reduced intracellular potassium ion level (Wang et al., 2018). However, in the latter two experiments, although changes in NLRP3 expression levels were confirmed, changes in GSDMD were not detected. All in all, it can be seen that the over-activation of pyroptosis has a significant impact on podocyte injury in DN. However, more studies are needed to confirm the activation mechanism of pyroptosis by different signaling pathways as described before, and we also need to find more effective therapeutic targets.

### Pyroptosis of renal tubular epithelial cells in diabetic nephropathy

From developmental studies, we have learned that the effect of renal tubular epithelial cells injury on DN seems to be no less than that of glomerular cells injury. DN is a syndrome characterized by renal ischemia and hypoxia, and renal tubular epithelial cells are very sensitive to hypoxia. Moreover, the exacerbation of renal tubular interstitial fibrosis will eventually lead to irreversible renal damage. Consequently, the study on the mechanism of renal tubular epithelial cells injury has been paid more and more attention in recent years. Some studies have found that when renal tubular epithelial cells are exposed to excessive molybdenum or cadmium, they can not only induce autophagy, but also activate the ROS/NLRP3/caspase-1 signaling pathway and lead to pyroptosis (Wei et al., 2020; Zhang et al., 2021b). Moretti J et al. found that caspase-11 can promote GSDMD cleavage and membrane translocation in renal tubular cells in AKI, and scanning electron microscope (SEM) results further confirmed the formation of small protrusion bodies (indicating pore formation in the membrane) (Miao et al., 2019). Previous studies have also shown that the activation of TNF-α/caspase-3/GSDME axis in ureteral can lead to pyroptosis and cell damage (Li et al., 2021j). When GSDME was inhibited, pyroptosis and the transcription of pro-inflammatory cytokines induced by AKI were alleviated (Xia et al., 2021). These evidences highlight the possibility of GSDMD or GSDME-mediated pyroptosis in renal tubular epithelial cells.

To investigate the mechanism of the NLRP3 inflammasome in DN tubular cells, Ding et al. found decreased miR-10a/b expression and increased NLRP3 inflammasome activation in db/db and STZ-treated mice. The reason may be related to the fact that miR-10a/b can target the 3′ untranslated region of NLRP3 mRNA and inhibit the assembly of NLRP3 inflammasome (Ding et al., 2021a). Furthermore, other studies have demonstrated NLRP3 inflammasome activation and pyroptosis in DN tubular cells. Wang et al. (2022d) found that miR-93-5p can also bind to NLRP3 and regulate pyroptosis of renal tubular epithelial cells in DN. At the same time, under the regulation of Wilms tumor 1-associating protein (WTAP), NLRP3 was methylated and pyroptosis was induced in HK-2 cells (Lan et al., 2022). Han et al. (2022) also found that overexpression of transcription factor EB (TFEB) can reduce the level of ROS and inhibit pyroptosis. Additionally, it was found that the cleavage of GSDME at 267–270 can be inhibited by inhibiting the activity of caspase-3 in STZ-induced diabetic mice, and tubulointerstitial fibrosis was improved (Wen et al., 2020). This result again demonstrated the role of the caspase-3/GSDME axis on tubular cell injury in DN. What’s more, some evidence emphasizes that lncRNA GAS5 and circACTR2, which function as molecular sponges, can affect the expression of NLRP3, caspase-1, GSDMD-N and IL-1β in renal tubular epithelial cells under HG conditions (Xie et al., 2019; El-Lateef et al., 2022). Vascular cell adhesion protein 1(VCAM1) has also been found to be positively correlated with pyroptosis and immune cell infiltration (Jia et al., 2021). All these evidences indicated that inhibition of pyroptosis has a protective effect on DN tubular cells. In addition, other studies have demonstrated that under HG conditions, the activation of NLRP3 inflammasome can be mediated by CD36 under and inhibited with OPTN by enhancing mitochondrial phagocytosis (Chen et al., 2019a; Hou et al., 2021). However, they did not examine whether the expression of GSDMD changed in the process. In short, pyroptosis in renal tubular epithelial cells of DN is regulated by different mechanisms, and inhibition of it has the potential to be a therapeutic target for slowing the progression of DN.

MicroRNAs (miRNAs) are a class of noncoding RNAs with potential roles in regulating the pathogenesis of various diseases. For instance, increased expression of miR-34a has been found in renal tubular epithelial cells from patients with renal fibrosis and mice with UUO (Liu et al., 2019c). The miR-21 in exosomes from renal tubular epithelial cells may accelerate the development of renal fibrosis through the PTEN/Akt axis (Zhao et al., 2021). Many studies have also reported the potential role of miRNAs in DN (Dewanjee and Bhattacharjee, 2018). For instance, miR-483-5p expression was reduced in HG-stimulated renal tubular epithelial cells, which attenuated its restriction on MAPK1 and TIMP2 mRNAs, ultimately promoting renal interstitial fibrosis (Liu et al., 2021b). In the STZ-induced diabetic mice, renal fibrosis was reduced by modulating the miR-21/Smad7 signaling pathway (Liu et al., 2019a). Furthermore, inhibition of miR-122-5p, miR-133b, and miR-199b can attenuate EMT of renal tubular epithelial cells in diabetic mice (Sun et al., 2018; Zang et al., 2022). Interestingly, miRNAs were also found to have regulatory roles of pyroptosis in tubular epithelial cells. For example, regulation of ANRIL/miR-497/TXNIP and miR-667-5p/NLRC4 axis can both promote the progression of pyroptosis in DN (Wang and Zhao, 2021; Li et al., 2022c). Collectively, different miRNAs may be potential strategies for the treatment of EMT and pyroptosis of renal tubular epithelial cells in DN.

### Pyroptosis of glomerular endothelial cells in diabetic nephropathy

Glomerular endothelial cells (GECs) are more susceptible to damage by circulating substances in the blood as the first barrier of the glomerular filtration membrane. Meanwhile, GECs are rich in mitochondria and require a lot of energy. Impaired GECs can affect hemodynamic and are closely related to the production of proteinuria. Moreover, renal tubular epithelial cells can release cytokines through autocrine or paracrine mechanisms and induce inflammatory responses to impair glomerular structure and function. Subsequently, damaged GECs can reduce blood supply to the renal tubules, leading to increased damage of renal tubules (Chen et al., 2020c). It can be seen that the effect of GECs damage on DN is also very important.

There is a close relationship between endothelial dysfunction and various diseases in humans. For example, in hyperuricemia, pyroptosis in human umbilical vein endothelial cells (HUVEC) can be promoted by regulating NLRP3 expression (Chi et al., 2021). The activation of the ROS/NLRP3/caspase-1 signaling pathway induced by oxidative stress can also lead to endothelial dysfunction and pyroptosis in chronic kidney disease (CKD) (Tang et al., 2019). Several studies have demonstrated that hyperglycemia can also adversely affect structure and function of GECs. For instance, in the DN mouse model, the overexpression of METTL14 induced by HG can promote apoptosis and inflammation in GECs (Li et al., 2021d). Moreover, loss of autophagy in endothelial cells altered the phenotype of GECs and led to sparse capillaries in the glomeruli of diabetic animals (Lenoir et al., 2015). It is worth noting that some recent studies have shown that the pyroptosis also exists in GECs. For instance, Han et al. (2021) regulated GSDMD expression by inhibiting interferon regulatory factor 2 (Irf2), which improved endothelial pyroptosis in DN. The use of NaB can also ameliorate HG-induced GECs damage by modulating the canonical inflammasome pathway *via* the NF-κB/IκB-α signaling pathway (Gu et al., 2019). However, there are still few studies on the role of NLRP3 inflammasome-mediated pyroptosis in GECs, and further research is needed.

## Epithelial-to-mesenchymal transition and endothelial-to-mesenchymal transition and pyroptosis in diabetic nephropathy

DN is histologically characterized by the excessive deposition of extracellular matrix (ECM) in glomerular mesangium, GBM, and tubulointerstitium (Steffes et al., 1989; Mason and Wahab, 2003). It has been suggested that activated fibroblasts/myofibroblasts are the main cells for the accumulation of ECM and that both epithelial-to-mesenchymal transition (EMT) and endothelial-to-mesenchymal transition (EndMT) can lead to the increase of fibroblasts (Kalluri and Neilson, 2003; Potenta et al., 2008). Numerous studies have shown that EMT in renal tubular epithelial cells is a key process in tubulointerstitial fibrosis (TIF) and also one of the key reasons for the progression of renal fibrosis in DN (LeBleu et al., 2013; Lovisa et al., 2015). Rather than being directly transformed into myofibroblasts, renal epithelial cells secrete cytokines and chemokines to promote the development of fibrosis (Carew et al., 2012). When EMT occurs, cell adhesion molecules, including E-cadherin and the zonula occludens (ZO-1) protein-1, are lost and replaced by the mesenchymal marker alpha-smooth muscle actin (α-SMA) and the intermediate filament proteins (Sun et al., 2020). EndMT refers to the transition from endothelial cells (ECs) to mesenchymal cells and can be considered as a special type of EMT that occurs in ECs. When EndMT occurs, the endothelial phenotypes are lost and mesenchymal-like characteristics are acquired in endothelial cells (Liang et al., 2016). In DN, EMT can occur in renal tubular epithelial cells and podocytes, while EndMT can occur in glomerular endothelial cells (Loeffler and Wolf, 2015; Tu et al., 2019; Shi et al., 2020). Inhibition of EMT or EndMT in renal cells can alleviate renal fibrosis in DN (Liu et al., 2016a; Shang et al., 2017).

Connective tissue growth factor (CTGF/CCN2), TGF-β1, IL-6, and sonic hedgehog (SHH) are all key regulators of renal fibrosis (Lovisa et al., 2015). Although there are many pro-fibrotic factors can affect renal function, the TGF-β/Smad signaling pathway is considered to be one of the major pathways that orchestrate renal fibrosis (Zheng et al., 2021). Numerous studies have shown that TGF-β can induce EMT and EndMT in renal cells of DN (Wang et al., 2014; Sun et al., 2018; Guan et al., 2022). Sustained HG levels induce the expression of TGF-β, which subsequently activates Smad 2/3 through phosphorylation. Inhibiting p53-mediated nuclear translocation of Smad2/3 may be an effective strategy to prevent diabetes-induced renal fibrosis (Higgins et al., 2018). Interestingly, Zhang et al. (2019c) found that silencing of GSDMD significantly reduced TGF-β expression in fibroblast-like synoviocytes. Moreover, Li et al. (2022b) also demonstrated in HG-induced renal tubular epithelial cells that regulation of TGFB1 can regulate pyroptosis and inhibit cellular inflammation and cell death. Although these studies suggest that the TGF-β signaling pathway may have a regulatory role in pyroptosis of DN, the specific mechanism still needs to be confirmed by more studies.

The Wnt/*β*-Catenin signaling pathway has also been shown to regulate EMT and EndMT in DN kidney cells (Li et al., 2015; Zhang et al., 2019b). However, there are two opposing views on the role of Wnt/*β*-Catenin axis in DN. It has been suggested that under conditions of high glucose or diabetes, the decreased secretion of some Wnt proteins can induce apoptosis and EMT in mesangial cells (Lin et al., 2006; Lin et al., 2008; Beaton et al., 2016). However, it has also been suggested that nitric oxide (NO) donor treatment can inhibit diabetes-mediated oxidative stress and reduce the expression of TGF-β1 (Hsu et al., 2015). Notably, it was found in breast cancer cells that inhibition of the wnt/*β*-Catenin signaling pathway can inhibite EMT and the expression of NLRP3 and IL-1β (Zheng et al., 2020). The use of siRNA or drugs to inhibit the expression of *β*-Catenin can inhibit the activation of NLRP3 inflammasome, which may be related to the fact that *β*-Catenin can interact with NLRP3 and promote the association of NLRP3 with ASC (Huang et al., 2020b). Furthermore, inhibiting the expression of TXNIP can also inhibit the Wnt/*β*-Catenin signaling pathway (Dong et al., 2020). Meanwhile, TXNIP can regulate NLRP3-mediated pyroptosis as previously described. Collectively, these studies suggest that the wnt/*β*-Catenin signaling pathway may have a regulatory effect on NLRP3, but no studies have directly demonstrated the role of the Wnt/*β*-Catenin axis in renal cells pyroptosis of DN.

Glucocorticoid receptor (GR), presenting in almost every tissue of the body, is a nuclear hormone receptor and the target of a number of synthetic steroids (Goodwin et al., 2013). The expression of *α*-SMA and *β*-Catenin were significantly increased after endothelial GR knockout in diabetic mice, and then EndMT was induced by upregulating Wnt signaling (Srivastava et al., 2021c). Additionally, loss of podocyte GR can also lead to the upregulation of Wnt signaling, the disruption of fatty acid metabolism, and the exacerbated glomerular fibrosis (Srivastava et al., 2021b). GR has also been shown to be a inducer of EMT in breast cancer cells (Shi et al., 2019), and overexpression of the homeobox protein HOX-A13 (HOXA13) in renal tubular epithelial cells can inhibit EMT by activating GR signaling (Peng et al., 2016). However, the regulation effect of EMT has not been demonstrated in DN kidney cells. Interestingly, studies have shown that inhibition of CASP1/NLRP3 expression increased GR levels (Paugh et al., 2015), and glucocorticoids can activate NF-κB/NLRP3 signaling pathway through GR (Feng et al., 2019). However, whether GR is involved in NLRP3-mediated pyroptosis in DN is still unclear.

Fibroblast growth factor receptors (FGFRs) are tyrosine kinase receptors that mediate biological responses by binding to fibroblast growth factors (FGFs) (Itoh and Ornitz, 2004). Specific knockout of FGFR1 in endothelial cells can lead to the activation of TGF-β signaling and exacerbate EndMT (Chen et al., 2014). Li et al. (2020b) also demonstrated that FGFR1 was a critical regulator of EndMT-associated EMT activation in diabetic kidneys in diabetic kidneys. FGF21, a potential diabetes drug, can inhibit the phosphorylation of tyrosine kinase *via* FGFR1. And tyrosine kinase can regulate the activation of NLRP3 inflammasome through phosphorylation of ASC. FGF21 can also significantly inhibit the expression of the expression of caspase-1 and IL-1β and improve vascular intimal hyperplasia in diabetic mice (Wei et al., 2019). FGFR1 has also been implicated in the PI3K/Akt signaling pathway, and knockdown of FGFR1 reduced the expression of TLR4 and NLRP3 in periodontitis (Huang et al., 2021a). However, whether FGFR is also involved in NLRP3-mediated pyroptosis in DN remains unclear.

Notch signaling is activated by interaction between Notch receptors and their ligands, which is a common mechanism of proteinuria in kidney disease (Murea et al., 2010). The expression of snail1 is directly regulated by the Notch signaling pathway, and the Notch/snail signaling pathway has been shown to regulate HG-induced EMT in renal tubular epithelial cells (Yang et al., 2017). In HUVECs, inhibition of Notch signaling can also significantly attenuate TGF-β1-induced EndMT (Yang et al., 2020). However, the regulatory effect of Notch signaling on EndMT in DN kidney has not been precisely studied. In addition to regulating EMT and EndMT, Notch1 signaling has also been reported to promote the activation of Snail and inhibit NLRP3 function in models of hepatic injury (Jin et al., 2020). Silencing of Notch1 in keloid fibroblasts fibroblasts also markedly inhibited the expression of the NLRP3 inflammasome and *α*-SMA (Lee et al., 2020). These findings suggest a possible regulatory role of Notch signaling on NLRP3, but whether it regulates pyroptosis in DN remains unclear.

The hedgehog interacting protein (Hhip) is a signaling molecule in the hedgehog pathway whose expression is quiescent after birth (Bishop et al., 2009). The activation of hedgehog signaling has been shown to be associated with EMT in liver cancer cells (Ding et al., 2017). In the UUO model, inhibition of hedgehog signaling pathway was also demonstrated to improve EMT in HK-2 cells (Li et al., 2021c). In the DN model, Zhao et al. found that hyperglycemia stimulated the expression of the Hhip by enhancing the generation of ROS, and TGF-β1/Smad2 pathway was also activated, which promoted the transition of glomerular endothelial cells to the mesenchyme. In addition, they found that Hhip expression was also increased in mouse podocytes cultured in a HG environment, resulting in podocyte loss and the activation of TGF-β1 and α-SMA (Zhao et al., 2018b). Furthermore, the use of the hedgehog inhibitor GANT-61 can also attenuate the expression of caspase-1, IL-1β, and IL-18 in chondrocyte (Liu et al., 2019b). However, there is no evidence to directly demonstrate the relationship between hedgehog and pyroptosis in DN.

Sirtuin3 (SIRT3) is a mitochondrial nicotinamide adenine dinucleotide (NAD+)-dependent deacetylase that can effectively prevent the development of DN, whether by regulating the AMPK/SIRT3 signaling pathway or the SIRT3/SOD2 signaling pathway (Liu et al., 2019d; Guan et al., 2021; Wongmekiat et al., 2021; Li et al., 2022a). Under HG conditions, the expression of SIRT3 was inhibited in HK-2 cells, and glycolysis was abnormally altered, ultimately leading to EMT (Li et al., 2020c). Srivastava et al. (2021a) also constructed endothelial SIRT3 knockout mice model and found that loss of SIRT3 accelerated EndMT in diabetic kidneys. Mechanistically, SIRT3 in endothelial cells can regulate glucose and lipid metabolism and mesenchymal transdifferentiation by regulating TGF-β/Smad3 axis. These evidences suggest that SIRT3 has a key role in diabetic renal fibrosis. In addition to regulating the process of renal fibrosis, SIRT3 has also been found to regulate the activation of inflammasomes. Guan et al. (2021) found that SIRT3 interacted with NLRC4 to promote its activation in macrophages, and SIRT3 is indispensable for the activation of the NLRP3 inflammasome. However, Liu et al. (2018) suggest that NLRP3 inflammasome activation was increased in SIRT3-deficient macrophages. In addition, studies have shown that cardiomyocyte pyroptosis can be reduced by activating the Nrf2/SIRT3 signaling pathway (Gu et al., 2021). However, there is no direct evidence that the role of SIRT3 in regulating NLRP3 inflammasome activation and pyroptosis is also present in DN kidney cells.

Dipeptidyl peptidase-4 (DPP-4) is a cell surface serine protease that cleaves various substrates, while dipeptidyl peptidase-4 inhibitors (DPP-4i) have the ability to inhibit DPP-4 enzyme activity under diabetic conditions. Therefore, DPP-4 inhibitors have been developed as novel agents for glycemic control in the clinic (Muskiet et al., 2014). Inhibition of DPP-4 activity can improve insulin sensitivity and reduce angiotensin II receptor-1 (AT-1) -mediated tubular-interstitial EMT (Huang et al., 2016). Reduction of DPP-4 expression in podocytes of diabetic rat can restore stromal cell-derived factor 1 α (SDF-1α) levels and may attenuate EMT through the activation of the PKA pathway (Chang et al., 2017). Additionally, linagliptin can also inhibit EndMT by inhibiting the activity of DPP-4, and improve renal fibrosis in STZ-induced diabetic mice. These evidences suggest that DPP-4 can also regulate EMT and EndMT. Furthermore, the use of linagliptin can reduce the expression of ASC, NALP3 and IL-1β in cardiomyocytes of db/db mice (Birnbaum et al., 2019). DPP-4i sitagliptin and omarigliptin can also inhibit NLRP3 expression in macrophages and HG-induced human glomerular endothelial cells (Dai et al., 2014; Li et al., 2021b). However, whether DPP-4i can also inhibit NLRP3-mediated pyroptosis in DN kidney cells remains to be verified.

## Pyroptosis and drugs in diabetic nephropathy

Because the complex pathogenesis of DN is not completely clear, the current treatment methods are still not enough to effectively delay the progression of DN. More and more evidences indicated that pyroptosis is one of the mechanisms of cell injury in DN. Therefore, therapeutic drugs targeting pyroptosis and their mechanism are worth exploring.

### Therapeutic drugs targeting NLRP3 inflammasome

One of the most characteristic of pyroptosis is activation of inflammasome. BAY 11–7082 is an NF-κB inhibitor that can inhibit NLRP3 inflammasome activation by inhibiting NLRP3-ATPase activity, and this effect is independent of its inhibitory effect on the NF-κB pathway (Juliana et al., 2010). Multiple studies have shown that BAY 11–7082 can reverse the activation of NLRP3 inflammasome-mediated pyroptosis, although this has not been validated in DN kidney cells (Qiu et al., 2017; Qiu et al., 2019). Meanwhile, the expressions of NLRP3, caspase-1 and IL-1β were reduced in db/db mice and HG-induced mesangial cells by the selective NLRP3 inhibitor MCC950. However, body weight and blood glucose levels were not affected (Zhang et al., 2019a). Moreover, it has been confirmed that the use of MCC950 can reduce the expression of pyroptosis-related proteins such as GSDMD in HG-stimulated podocytes (Liu et al., 2021a). Wang et al. also found that after Fucoidan (FPS) treatment, AMPK/mammalian target of rapamycin complex 1 (mTORC1)/NLRP3 signaling axis was regulated and podocyte pyroptosis was inhibited in DN rats (Wang et al., 2022a). Geniposide (GE) can effectively reduce the expression of NLRP3, cleaved-caspase-1 and GSDMD-N in HG-induced podocytes, and the mechanism may be related to the APMK/SIRT1/NF-κB signaling pathway (Li et al., 2020a). Another study found that the effect of saxagliptin in delaying the progression of DN also seems to be related to the inhibition of NLRP3 inflammasome activation (Birnbaum et al., 2016). Additionally, the glucagon-like peptide-1 analog liraglutide, a drug that can reduce the risk of adverse renal outcomes in diabetic patients, can also inhibit NLRP3-mediated pyroptosis in cardiomyocytes (Chen et al., 2018a). Furthermore, the administration of quercetin and allopurinol to high-fat diet (HFD) and STZ-induced diabetic mice significantly reduced the expression of NLRP3, caspase-1, IL-1β, and IL-18, but their effects on pyroptosis still require more experimental validation (Wang et al., 2012). Recently, the experiments in other cells have also found that Kuijieling (KJL), rosuvastatin (RVS), vitamin D (VD), and Kanglexin (KLX) can also inhibit NLRP3 inflammasome-mediated pyroptosis, but whether these drugs act on pyroptosis in DN kidney is still lacking specific research (Bian et al., 2020b; Chen et al., 2021; Zhang et al., 2021f; Jie et al., 2021). Overall, some of the above studies have demonstrated the inhibitory effect of certain drugs on NLRP3-mediated pyroptosis in DN kidney cells, but some only demonstrated the inhibitory effect of drugs on NLRP3 inflammasome activation, and did not detect pyroptosis. However, targeting the NLRP3 inflammasome to treat DN still has certain potential.

### Therapeutic drugs targeting reactive oxygen species

In DN, the imbalance of oxidation/antioxidant in renal cells will lead to excessive production of ROS and decreased expression of Nrf2 and other antioxidant factors, which can eventually lead to various forms of cell death. Punicalagin (PU) is a polyphenol that can reduce ROS production and exert antioxidant effects by promoting the production of SOD. The activation of TXNIP/NLRP3 axis was inhibited due to reduced ROS production, suggesting that this may be the mechanism by which PU inhibited pyroptosis of renal cells in diabetic mice (An et al., 2020). Besides, the pyroptosis induced by I/R was inhibited with salvianolic acid B (Sal B) by promoting the accumulation of Nrf2 through its antioxidant properties, although its effectiveness has not been demonstrated in DN (Pang et al., 2020). Since mitochondrial dysfunction is inseparable from the increase of ROS production, Yang et al. (2019b) treated DN mice with mitochondria-targeted peptide (MTP)-131/SS31, a mitochondria-targeted antioxidant peptide. The results showed that hydrogen peroxide (H2O2) and other free radicals were eliminated, accompanied by decreased expression of dynamin-related protein 1 (Drp1), caspase-1, and IL-1β, but whether GSDMD was cleaved in this process still needs further study. Moreover, Qu et al. (2022) demonstrated that pyrroloquinoline quinone (PQQ) can reduce mitochondrial dysfunction and ROS production, and improved renal fibrosis induced by hyperglycemia. In addition, Gao et al. (2022b) restored mitochondrial morphology by culturing podocytes with sialic acid precursor N-acetylmannosamine (ManNAc) and inhibited HG-induced pyroptosis by ROS/NLRP3 signaling pathway. These evidences suggested that DN can be improved by inhibiting pyroptosis by ameliorating mitochondrial dysfunction or reducing ROS production.

### Therapeutic drugs targeting caspase1/GSDMD

GSDMD is the most frequently studied protein with pore-forming characteristic that can be cleaved by cleaved-caspase-1. In the treatment of multiple sclerosis, when GSDMD reacts with dimethyl fumarate (DMF) to lead to succination, its reaction with caspase-1 will be restricted and pyroptosis will be inhibited (Humphries et al., 2020). Targeting caspase1/GSDMD in DN has also achieved good results. Han et al. (2021) observed amelioration of renal injury in stz-induced diabetic mice after injecting with hirudin, which may be related to the inhibition of Irf2/GSDMD axis. As an inhibitor of caspase-1, Vx-765 can improve the dysfunction of renal tubular epithelial cells in DN and regulate pyroptosis without affecting blood glucose levels or body weight (Wen et al., 2022). Other studies have shown that carnosine can inhibit HG-induced podocyte pyroptosis through its target caspase-1 (Zhu et al., 2021). Additionally, Sodium butyrate (NaB) can also inhibit pyroptosis by inhibiting caspase1/GSDMD axis in HG induced glomerular endothelial cells (GECs) (Gu et al., 2019). Taken together, these evidences suggested that caspase1/GSDMD axis may serve as one of the therapeutic targets for DN.

### Chinese proprietary medicine targeting pyroptosis in diabetic nephropathy

Due to the limitations of current drugs for the treatment of DN, the therapeutic effect of the Chinese proprietary medicine (CPM) has also begun to attract widespread attention. It has been reported that ginsenoside compound K (CK) with hypoglycemic effect can not only inhibit the activation of TXNIP/NLRP3 signaling pathway and the production of IL-1β and IL-18, but also reduce blood glucose, serum creatinine, and 24-h urine protein of the DN mice (Song et al., 2018b). Meanwhile, the activation of NLRP3 inflammasome and pyroptosis can be inhibited by ginsenoside Rg1 in podocytes of diabetic mice *via* mTOR/NF-κB/NLRP3 axis (Wang et al., 2020d). Additionally, ginsenoside Rg5 can also significantly inhibit the activation of NF-κB/NLRP3 axis and the phosphorylation of the three subfamilies of MAPK (Zhu et al., 2020). However, although they demonstrated inhibition of NLRP3 by these drugs, whether this renoprotective effect is also related to NLRP3 inflammasome-mediated pyroptosis remains to be verified. Furthermore, GSDMD-dependent pyroptosis can be inhibited by regulating TGF-β1 in HK-2 cells treated with Tanshinone IIA (Li et al., 2022b). Tangshen formula (TSF) can inhibit pyroptosis by regulating the TXNIP-NLRP3-GSDMD signaling pathway in HK-2 cells. Yi Shen Pai Du Formula (YSPDF) can regulate the Nrf2/HO-1 signaling pathway and reduces the generation of ROS and the expression of NLRP3, ASC, and caspase-1 in DN (Li et al., 2020d; Zhang et al., 2021d). Recently, it was also found that tetrahydroxy stilbene glucoside (TSG), the total flavonoids of Astragalus (TFA), Huangkwai capsule (HKC), and artificially cultivated Ophiocordyceps sinensis (ACOS) could improve kidney injury in DN. Their therapeutic mechanism may be related to the activation of PTEN/PI3K/Akt axis, TLR4/NF-κB axis, and the activation of NLRP3 inflammasome associated with P2X7R (Li et al., 2018a; Wang et al., 2018; Han et al., 2019; Liu et al., 2021a). Although some of these drugs only demonstrated an effect on NLRP3 inflammasome or caspase family and not indicated whether other pyroptosis markers such as GSDMD were also affected in the processes, the use of CPM to treat DN by inhibiting pyroptosis still has a lot of research value. Supplementary Table S1 summarized the drugs mentioned above related to NLRP3 inflammasome and pyroptosis.

### Potential drugs that have been evaluated for diabetic nephropathy treatment

Abnormal glucose metabolism also plays a contributing role in the development of DN. Impaired glycolysis may lead to disturbance of podocyte energy supply in DN and affect podocyte cytoskeletal structure (Luo et al., 2022). It has been reported that glucose fluctuation (GF) can also induce kidney injury, and regulation of HIF-1α/miR-210/ISCU/FeS signaling pathway can antagonize GF-induced kidney damage in glomerular mesangial cells (GMCs) by regulating aerobic glycolysis (Xu et al., 2021a). It has also been shown that glycolysis also plays a key role in pyroptosis of LPS-stimulated macrophages, and glycolysis inhibitors can inhibit HIF-1α downregulation and pyroptosis (Aki et al., 2020). Moreover, the use of glycolysis inhibitor 2-deoxy-D-glucose (2-DG) can also inhibit LPS-induced pyroptosis in microglial (Li et al., 2021e). However, the role of glycolysis inhibitors in pyroptosis of DN has not yet been demonstrated. SIRT3 is a major mitochondrial deacetylase involved in the activation of many oxidative pathways. In clinical trials and the diabetic animal models, Li et al. found that SIRT3 deficiency can promote abnormal glycolysis and HIF-1α accumulation, and the abnormal glycolysis is associated with the increased mesenchymal transition rate (Srivastava et al., 2018). Resveratrol (RES) has the effect of activating SIRT3 and can effectively reduce blood sugar levels without any side effects, and RES can also reduce urinary albumin excretion in DN patients (Sattarinezhad et al., 2019; Gowd et al., 2020). Moreover, RES can promote mitophagy to inhibit NLRP3 inflammasome activation and can inhibit pyroptosis through SIRT1 (Chou et al., 2019; Fan et al., 2021)**.** Honokiol (HKL) is also an activator of SIRT3 and can inhibit the expression of NLRP3, caspase-1, GSDMD by activating Nrf2 in human bronchial epithelial cells (Liu et al., 2021c). Although these evidences suggest that regulation of SIRT3 has a protective effect on cells and may also regulate NLRP3 inflammasome-mediated pyroptosis, there is no direct evidence for the role of SIRT3 activators in pyroptosis of DN. DPP-4 is a member of serine proteases and plays a major role in glucose metabolism. DPP-4 inhibitors are a class of antidiabetic drugs and have nephroprotective properties (Gupta and Sen, 2019). Saxagliptin and linagliptin are both DPP-4 inhibitors and both have the potential to reduce proteinuria in patients with type 2 diabetes (Groop et al., 2015; Mosenzon et al., 2017). In db/db mice, linagliptin can also inhibit renal inflammation and fibrosis induced by C-reactive protein (CRP)/CD32b/NF-κB axis (Tang et al., 2021). Interestingly, studies have found that linagliptin can reduce the expression of ASC, NLRP3, IL-1β, TNF-α, and inhibit apoptosis in db/db mice, and this effect is dependent on the activation of p38 and the inhibition of TLR4 expression (Birnbaum et al., 2019). But another study found that DPP-4i not only activated the ROS/NRF2/HO-1 axis in breast cancer cells, but also triggered ROS-dependent NF-κB activation. Moreover, DPP-4i also triggered ROS/NF-κB-dependent NLRP3 inflammasome activation (Li et al., 2021f). Although no studies have shown the relationship between DPP-4i and NLRP3 inflammasome-mediated pyroptosis in DN, these evidences may provide new insights into the unexpected side effects of DPP-4i in diabetic patients with other diseases.

Early stages of DN are characterized by elevated glomerular filtration rate (GFR) and increased filtration fraction (FF) (Hannedouche et al., 1990; Komers et al., 2011). The RhoA/ROCK axis plays a role in the control of vascular tone in the kidney. The Rho-associated kinases (ROCK) inhibitors Y27632 and fasudil were examined to have renoprotective effects on DN (Komers et al., 2011). Fasudil also ameliorated albuminuria and glomerular hypertrophy in DN mice by downregulating HIF-1α expression (Matoba et al., 2013). Furthermore, the application of fasudil can reduce proteinuria and improve renal prognosis by inhibiting ROCK activity in diabetic patients (Matoba et al., 2021). Studies have also shown that fasudil can inhibit NF-κB nuclear translocation and TGF-β1 expression in STZ-induced diabetic rats (Xie et al., 2013). ROCK Inhibitor-Y27632 can also reverse the expression of NF-κB, NLRP3, ASC, and caspase-1 induced by ventilator-induced lung injury (VILI) (Zhang et al., 2021e). However, studies on whether ROCK inhibitors also have an effect on NLRP3-mediated pyroptosis are still lacking. Mineralocorticoid receptor (MR) has a role in regulating the transcription of target genes, and both elevated aldosterone levels and MR hyperactivation can lead to salt and water retention and hypertension (Tirosh et al., 2010). Mineralocorticoid receptor antagonists (MRA) also have therapeutic effects on eGFR, proteinuria, and hyperkalemia in diabetic rats and patients with CKD (Bhuiyan et al., 2019; Baran et al., 2021; Patel et al., 2021). ACE inhibitors (ACEIs) and AT1 receptor antagonists (ARBs) are thought to reduce the progression of ESRD in diabetic patients, and their effects have been extensively validated in DN patients and mouse models (Zheng et al., 2006; Nakagawa, 2010; Tesch et al., 2019). In clinical trials, the combination of MRA and ACEI/ARB significantly reduced urinary albumin excretion and urinary albumin-creatinine ratio and significantly increased the risk of hyperkalemia (Sun et al., 2017). Furthermore, N-acetyl-seryl-aspartyl-lysyl-proline (AcSDKP) is considered to be one of many anti-fibrotic molecules that ACE inhibitors exert their anti-fibrotic effects, and the inhibition of AcSDKP can lead to the activation of mesenchymal transition and renal fibrosis in diabetic mice (Castoldi et al., 2013; Nitta et al., 2016; Srivastava et al., 2020). Studies have also shown that AcSDKP can also predict changes in renal function in normoproteinuric diabetic patients (Nitta et al., 2019), and AcSDKP levels are also associated with sodium intake (Kwakernaak et al., 2013). These findings provide many clues for the anti-fibrotic effect of AcSDKP in human kidney disease. Interestingly, one study demonstrated that the activation of MR was involved in the NLRP3/caspase-1 axis-induced pyroptosis of UUO model (Ma et al., 2019). Studies have also shown that AcSDKP was found to increase Akt phosphorylation in cancer cells (Hu et al., 2013). Meanwhile, we have previously described the potential impact of the PI3K/Akt signaling pathway on pyroptosis. Taking together, although the above-mentioned drugs have been used clinically, there are still many gaps in the research on their mechanism.

## Future directions and perspective in diabetic nephropathy

Many of the studies discussed above clearly demonstrated that NLRP3-dependent pyroptosis occurs during the progression of DN, and the inhibition of NLRP3 inflammasome activation through different signaling pathways can inhibit pyroptosis and ameliorate renal injury. However, several remaining issues must be addressed. First, NLRP3 inflammasome activation does not necessarily lead to pyroptosis, so are the protective effects of some NLRP3 inflammasome inhibitors related to the inhibition of pyroptosis? What conditions can trigger NLRP3 inflammasome-mediated pyroptosis? Second, whether some of our proposed signaling pathways can trigger pyroptosis by activating the expression of NLRP3 in DN kidneys is still lacking in specific studies. Meanwhile, are there other signaling pathways that can also activate NLRP3 inflammasome assembly and trigger pyroptosis? Furthermore, EMT and EndMT also appear to be linked to pyroptosis. Therefore, can exploring this potential link also provide new perspectives for DN treatment and find more effective therapeutic targets? Finally, many drugs have been discovered to have therapeutic effects on DN, and they can inhibit the expression of NLRP3. However, studies are still lacking to show whether their mechanisms are also related to pyroptosis. May further investigation of the mechanism of these drugs and their possible side effects on some concomitant diseases provide more effective options for clinical application?

## Conclusion

In this review, we summarized three mechanisms of pyroptosis and discussed the relationship between pyroptosis and NLRP3 inflammasome activation. In addition, we explored several pathways related to NLRP3 inflammasome activation, involving NF-κB, TXNIP, Nrf2, PI3K/Akt and other important signaling molecules. These pathways are linked to each other, complicating the activation mechanisms of NLRP3 inflammasome. However, the inhibition of NLRP3 inflammasome, caspase-1, GSDMD and other proteins in pyroptosis can alleviate the kidney damage in DN. In conclusion, the research on the mechanism of NLRP3 inflammasome-mediated pyroptosis in DN is still ongoing, and more effective drugs are expected to be found.
