# Supplementary material for: NLRP3-mediated pyroptosis in diabetic nephropathy
Source: Front Pharmacol. 2022 Oct 11;13:998574. doi: 10.3389/fphar.2022.998574 (PMC9593054; doi:10.3389/fphar.2022.998574)
Supplement: Supplementary file 1 [file Table1.DOCX]

Supplementary Material

# Supplementary Tables

**Table S1.** Therapeutic drugs targeting NLRP3-mediated pyroptosis

| **Types** | **Drugs** | **Animals or cells** | **Effects** |
| --- | --- | --- | --- |
| Drugs on pyroptosis in DN | Fucoidan (FPS) | DN model rats and Podocytes | Regulating the AMPK/mTORC1/NLRP3 signaling axis |
|  | Geniposide (GE) | HFD/STZ-induced DN mice and Podocytes | Regulating the APMK/SIRT1/NF-κB pathway and the expression of NLRP3 |
|  | Punicalagin | HFD/STZ-induced DN mice | Downregulating the expression of NOX4 and inhibiting the TXNIP/NLRP3 axis |
|  | ManNAc | STZ-induced DN mice and Podocytes | Inhibiting mitochondrial injury and the ROS/NLRP3 signaling pathway |
|  | Hirudin | STZ-induced DN mice | Ameliorated GSDMD-mediated pyroptosis by inhibiting IRF2 |
|  | VX-765 | HK-2 cells | A selective caspase-1 inhibitor |
|  | Carnosine | STZ-induced DN mice and MPC5 cells | Inhibiting pyroptosis via the targeting of caspase-1 |
|  | Sodium butyrate (NaB) | Renal glomerular endothelial cells | The NF-κB inhibitor (IκB-α) and inhibiting the NF-κB/NLRP3 axis |
|  | Ginsenoside Rg1 | Diabetic rat snd Podocytes | Inhibiting pyroptosis through the mTOR/NF-κB/NLRP3 axis |
|  | Tanshinone IIA | HK-2 | Regulating the TGF-β1-dependent pathway and the expression of NLRP3 |
|  | Tangshen formula (TSF) | STZ-induced DN mice and HK-2 cells | Regulating the expression of ROS and TXNIP/NLRP3/GSDMD axis |
|  | Yi Shen Pai Du Formula (YSPDF) | The db/db mice and HK-2 cells | Regulating the activation of Nrf2 and the NLRP3 inflammasome, inhibiting the oxidative stress and EMT |
|  | The total flavones of Abelmoschus manihot (TFA) | Podocytes | adjusting METTL3-dependent m6A modification and regulating NLRP3-inflammasome activation and PTEN/PI3K/Akt signaling. |
|  | MCC950 | Renal glomerular mesangial cells and podocytes | a selective and potent inhibitor of NLRP3 |
| Drugs associated with NLRP3 in DN | Quercetin | STZ-induced DN Rats | Inhibiting the NLPR3/caspase-1/IL-1β pathway |
|  | Allopurinol | STZ-induced DN Rats | Inhibiting the NLPR3/caspase-1/IL-1β pathway |
|  | Saxagliptin | BTBR (T2DM) and Akita (T1DM) mice | Inhibiting the expression of NLRP3, TNF-α and caspase-1 |
|  | Maresin 1 | Renal glomerular mesangial cells | Inhibiting the expression of NLRP3、caspase-1 and IL-1β |
|  | Mitochondria-targeted peptide SS31 | STZ-induced DN mice and HK-2 cells | Suppressing the expression of Drp1 and increasing the expression of Mfn1, decreasing the mitochondrial fragmentation |
|  | Pyrroloquinoline quinone (PQQ) | STZ-induced DN mice | Inhibiting the expression of NF-κB, ROS, NLRP3, caspase-1, IL-1β |
|  | Ginsenoside compound K (CK) | Renal glomerular mesangial cell line HBZY-1 | Inhibiting the ROS-mediated activation of NLRP3 inflammasome and NF-κB/p38 signaling pathway, |
|  | Ginsenoside Rg5 | HFD/STZ-induced DN mice | Inhibiting the expression of NF-κB, NLRP3, caspase-1, IL-1β and the phosphorylation of p38 MAPK |
|  | Huangkui capsule (HKC) | STZ-induced DN mice | Inhibiting the activation of NLRP3 inflammasome and the TLR4/NF-κB signaling pathway |
|  | Linagliptin | db/db mice | Inhibitor of dipeptidyl peptidase-4; inhibiting the expression of ASC, NLRP3, IL-1β and TNF-α |
|  | Artificially cultivated ophiocordyceps sinensis (ACOS) | STZ-induced DN rats and Podocytes | Inhibiting the expression of P2X7R and the activation of NLRP3 inflammasome |
|  | Tetrahydroxy stilbene glucoside (TSG) | Podocytes (MPC5) | Inhibiting the activation of NLRP3 inflammasome |
| Potential drugs on pyroptosis | Liraglutide | H9c2 cardiomyoblasts | Regulating the SIRT1/NOX4/ROS signaling pathway |
|  | Kuijieling (KJL) | Ulcerative colitis (UC) mouse model and RAW264.7 cells | Inhibiting the expression of NLRP3, ASC, caspase-1, miR-223 and GSDMD-N |
|  | Rosuvastatin (RVS) | Coronary microembolization (CME) mice and H9c2 cells | Inhibiting the expression of ROS, NLRP3, caspase-1, IL-1β and GSDMD-N |
|  | Vitamin D | Non-alcoholic fatty liver disease (NAFLD) rats and BRL-3A cells | Inhibiting the expression of NLRP3 Gasdermin D |
|  | Kanglexin (KLX) | (LPS)-treated mouse ventricular cardiomyocytes | Inhibiting the expression of NLRP3 GSDMD-N and cleaved - caspase-1 |
|  | Salvianolic acid B (SalB) | Renal tubular epithelial cells | Regulating the Nrf2/NLRP3 signaling pathway |
|  | Dimethyl fumarate (DMF) | Macrophages | Reacting with GSDMD, GSDMD succination prevents its interaction with caspases |
|  | 2-deoxy-D-glucose (2-DG) | microglial | 2-DG is a glycolysis inhibitor; LPS requires glycolysis to induce microglial pyroptosis. |
|  | Honokiol (HKL) | human bronchial epithelial cell line BEAS-2B | Inhibiting the expression of NLRP3, caspase-1, GSDMD by activating Nrf2 |
|  | Resveratrol (RES) | HK-2 cells | Activating SIRT1 and inhibiting NRLP3 inflammasome and pyroptosis |
| Potential drugs associated with NLRP3 | BAY 11-7082 | Macrophage | A phosphorylated NF-κB inhibitor (IκB), suppressing the activation of NLRP3 inflammasome |
|  | Fisetin | HK-2 cells | Inhibiting the expression of NLRP3 and NF-κB |
|  | Y27632 | alveolar macrophages (AMs) | Y27632 is a ROCK inhibitor; Inhibiting the expression of ASC, NLRP3, caspase-1 and NF-κB |

DN, Diabetic nephropathy; HFD/STZ, High fat diet and streptozotocin; AMPK, Adenosine 5’-monophosphate (AMP)-activated protein kinase; mTORC1, Mechanistic target of rapamycin complex 1; NLRP3, NOD-like receptor protein 3; SIRT1, Sirtuin 1; NF-κB, Nuclear-factor κB; NOX4, NADPH Oxidase 4; TXNIP, Thioredoxin-interacting proteins; ROS, Reactive oxygen species; GSDMD, Gasdermin D; IRF2, Interferon regulatory factor 2; HK-2, (human kidney 2), a proximal tubular cell line; MPC5, Mouse podocyte clone-5; IκB-α, Inhibitor of NF-κB; METTL3, Methyltransferase-like protein 3; EMT, Epithelial-mesenchymal transformation; PTEN, Phosphatase and tensin homologue; PI3K, Phosphatidylinositol-3-kinase; Akt, Serine/threonine-protein kinase; IL-1β, Interleukin-1 beta; TNF-α, Tumor necrosis factor alpha; Drp1, Dynamin-relatedprotein 1; Mfn1, Mitofusin 1; MAPK, Mitogen-activated protein kinases; TLR4, Toll-like receptors 4; P2X7R, Purinergic P2X7 receptor; SIRT1, Silencing message modulator related enzyme 1; ASC, Apoptosis-associated speck-like protein containing CARD; SIRT3, Sirtuin3; ROCK Rho associated kinases.
